# Functional 3D Human Neuron–Glioblastoma Model Reveals Cellular Interactions Enabling Drug Safety Assessments

**DOI:** 10.1096/fj.202500291RR

**Published:** 2025-04-25

**Authors:** Nanna Förster, Lotta Isosaari, Oskari Kulta, Oona Junnila, Valtteri Vuolanto, Marjukka Pollari, Kirsi J. Rautajoki, Susanna Narkilahti

**Affiliations:** ^1^ NeuroGroup, Faculty of Medicine and Health Technology Tampere University Tampere Finland; ^2^ Department of Oncology, Tays Cancer Center Tampere University Hospital Tampere Finland; ^3^ Cancer Regulation and Immunology Group, Faculty of Medicine and Health Technology Tampere University Tampere Finland; ^4^ Tays Cancer Centre Tampere University Hospital Tampere Finland

**Keywords:** brain tumor, cellular functionality, coculture, glioma, human induced pluripotent stem cell‐derived neurons, hydrogel, in vitro, temozolomide, tissue engineering

## Abstract

Glioblastoma (GB) cells actively interact with the central nervous system (CNS) tumor microenvironment (TME). These interactions, particularly with neurons, require a better understanding. 3D tumor models replicating the human TME are needed to unravel pathological processes and to test novel treatments for efficacy and safety. We developed a novel 3D human coculture model for studying neuron–GB interactions. The model revealed both structural and functional interactions between cell types. Paracrine communication in the coculture model favored a tumor‐supportive environment. Notably, cell‐specific calcium signaling characteristics differed in cocultures compared to monocultures, highlighting the impact of interactions on cellular functionality in TME. The safety of a clinically used treatment, temozolomide, was tested in the 3D coculture model, and it selectively inhibited GB invasion while preserving neurons' morphology and functionality. The established model provides a tool for dissecting the interactions within the TME and testing the efficacy and safety of novel treatments.

## Graphical abstract

A human 3D coculture model enabled the study of cellular interactions in glioblastoma (GB). Neurons formed direct connections with GB cells, while paracrine signaling promoted a tumor‐supportive environment. Functional interactions indicated bidirectional communication, altering the activity of both cell types. Model validation with temozolomide showed GB invasion inhibition while preserving neuronal morphology and function. This model serves as a tool for dissecting GB interactions and evaluating the efficacy and safety of novel treatments. Created in BioRender. Isosaari, L. (2025) https://BioRender.com/e790igg.

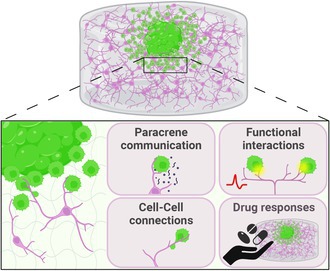

## 1 Introduction

Diffuse gliomas are primary brain tumors that are difficult to treat [1]. Glioblastoma (GB) is the most aggressive and common form, with an average survival time of only 15 months after diagnosis [2]. The standard of care for GB patients includes surgical resection followed by radiotherapy and chemotherapy with temozolomide (TMZ). Unfortunately, these treatments have poor clinical efficacy or result in high recurrence rates due to the infiltrative nature of the cancer cells and drug resistance of heterogeneous GBs [2]. Despite improvements in cancer research overall, GBs are still the most common cause of mortality from brain tumors and are among the most lethal cancers [3].

In vivo, GB cells reside in a specific niche in the central nervous system (CNS) called the tumor microenvironment (TME) which contains nontumor cells, such as neurons and astrocytes, stromal components, vascular elements, and extracellular matrix (ECM) molecules [4]. CNS‐GB crosstalk plays a key role in tumor development [5]. Importantly, neurons prompt glioma growth by electrochemical signals at neuron–glioma synapses [6, 7]. GB cells communicate with each other via intercellular calcium ion (Ca^2+^) waves [8], while neuronal input increased the Ca^2+^ signal frequency in high‐grade glioma cells in 2D in vitro cocultures and in vivo xenograft mouse models [6, 7, 9]. Conversely, GB cells can affect Ca^2+^ activity in neurons [10]. In addition, evidence of paracrine signaling between neurons and high‐grade glioma cells has been shown [11]. Taken together, there exists a possibility to obstruct neuron–glioma crosstalk to hinder tumor progression [12]. However, a more comprehensive understanding of these interactions at the functional level is needed to test both the safety and efficacy of different treatment strategies.

TME has an important role in brain cancer pathogenesis and treatment response [12]. Most of the current in vitro GB modeling approaches have focused on immunologic interactions in the TME [13, 14, 15]. Although traditional 2D cancer cell models have led to many important findings over the past century [16], they usually do not consider the influence of the TME. In contrast, 3D culture conditions offer the possibility to incorporate parts of the native TME into in vitro models. For instance, patient‐derived GB organoids imitate the hypoxic and heterogeneous characteristics of tumors [17], while human cerebral organoids cocultured with GB cells incorporate healthy brain cells around the tumor mass [18, 19]. However, organoids are laborious models with low throughput and lack ECM components. To study invasion and ECM influence, 3D hydrogel‐based models have been established [13, 20, 21], while coculture models are convenient to explore tumor–neuron interactions [5].

Despite the recent advancements, GB in vitro modeling requires further development. Namely, incorporating relevant cell types and ECM molecules into models, as well as the implementation of functional studies, is needed. For example, drug treatment experiments involving cocultures of GB and CNS cells usually assess drug effects only on cancer cells and ignore the responses of nonmalignant cells. However, treatment‐associated neurotoxicity is a critical issue that should be assessed more carefully in models [22]. Furthermore, cellular functionality has rarely been assessed in 3D in vitro GB models. Few studies have shown Ca^2+^ activity in 3D coculture systems, supporting the concept of bidirectional Ca^2+^ signaling, where GBs increase neuronal functionality and neurons promote Ca^2+^ oscillations in GB cells [20, 23]. The lack of human‐derived neurons in these studies hampers the translation of the results. Moreover, despite the critical role of functionality for GB cells, neurons, and their interactions, the assessment of cellular functionality has not been conducted in drug response studies utilizing human‐derived 3D in vitro GB models. Thus, there is a need for human cell‐derived models in which cellular functionality and drug safety and efficacy can be assessed in more detail.

In this work, we developed a physiologically relevant, hydrogel‐based 3D neuron–GB model that enables the assessment of tumor‐TME interactions with improved credibility. A coculture of hiPSC‐derived cortical neurons with human GB spheroids was established in 3D with a collagen I hydrogel. Immunocytochemical (ICC) staining, protein secretion analysis, and Ca^2+^ imaging were performed to assess physical interactions, bidirectional chemical, and electrical signaling between neurons and GB cells, and the repeatability of the model. Furthermore, drug responses to TMZ were evaluated in both GB cells and neurons to validate the model as a tool to assess both drug efficacy and safety.

## 2 Materials and Methods

### 2.1 Cells

#### 2.1.1 Human Induced Pluripotent Stem Cells

Two in‐house generated human induced pluripotent stem cell (hiPSC) lines (UTA.04511.WTs and UTA.10902.EURCCs [24]) and one commercial hiPSC line with a homogenous red fluorescent protein (RFP) tag (TUBA1B WTC, AICS‐0031‐035, Coriell Institute, Camden, New Jersey, USA) were used for cortical neuron differentiation. The pluripotency of the lines was confirmed regularly, and all cultures maintained normal karyotypes and were free of mycoplasma. The in‐house made lines were obtained from the Faculty of Medicine and Health Technology (MET), iPS Cells facility, Tampere University, Finland. The hiPSCs used were acquired from voluntary subjects who provided written informed consent. The project has a supportive statement from the Ethics Committee of the Expert Responsibility Area of Tampere University Hospital to use the named hPSC lines in neuronal research (R20159).

#### 2.1.2 GB Cells

The GB cell line LN229/GFP was purchased from FenicsBIO (CL‐1118, Halethorpe, Maryland, USA). The LN229/GFP line is derived from the human GBM cell line LN229 by integrating a transgenic turbo green fluorescent protein (GFP) gene for autofluorescence. LN229/GFP cells were cultured in LN229‐medium including DMEM (Thermo Fisher Scientific, Waltham, Massachusetts, USA) supplemented with 5% fetal bovine serum (FBS, Thermo Fisher Scientific, Waltham, Massachusetts, USA). LN229 was used for the studies because it is well characterized and has been used extensively in preclinical studies [21, 25].

### 2.2 Methods Details

#### 2.2.1 Experimental Design

**FIGURE 1:**
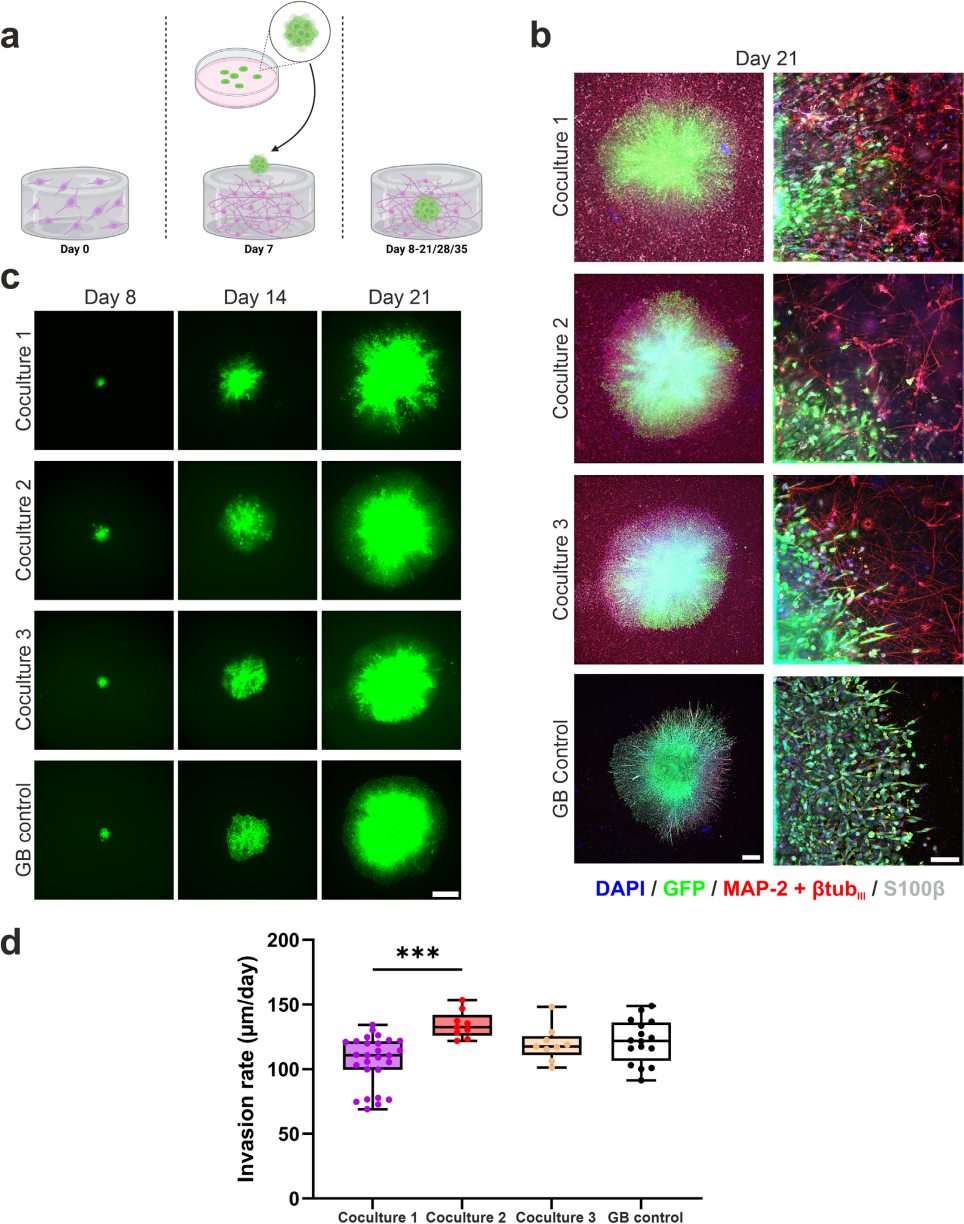
Establishment of long‐term 3D neuron‐GB model. (a) Schematic overview of the model setup. Cortical neurons were plated in collagen I hydrogel and grown for 7 days before the GB spheroids were embedded. Cocultures were maintained for 2–4 weeks. Created in BioRender. Isosaari, L. (2025) https://BioRender.com/v29d558. (b) ICC staining on Day 21 revealed successful model establishment which was repeatable with neurons derived from three different hiPSC lines. The cells remained viable, and the GB spheroids (labeled with GFP) invaded into the neuronal networks (MAP‐2 + ßtub_III_). Cell nuclei were stained with 4',6‐diamidino‐2‐phenylindole (DAPI). Scale bars are 500 μm (left column) and 100 μm (right column). (c) GB spheroid growth was followed during the coculture period, revealing steady invasion in all culture setups. The scale bar is 1000 μm. (d) Quantification of spheroid invasion rates (calculated from Days 8 to 21) revealed similar rates in cultures with neurons derived from different hiPSC lines (*n* = 27,9 and 9 for Coculture 1/2/3, respectively) and in GB monoculture (*n* = 19). Differences between groups were analyzed with the nonparametric Kruskal–Wallis test with Dunn's correction for multiple comparisons. Significance is indicated by *** < 0.001. See also Figure S1 and Video S1.

In this work, the aim was to develop a 3D human‐derived model of neuron–glioblastoma (GB) interactions, with the objectives of assessing both physical and functional interactions and determining the effect of a clinically used drug on the system. The model was established by embedding a GB spheroid in a preformed neuronal network in a collagen I hydrogel (Figure 1a) to better mimic the physiological occurrence of GB in vivo. The neuronal network and GB spheroids were further cocultured for 2–4 weeks, and the interactions were assessed via live cell imaging, immunocytochemical (ICC) staining, calcium ion (Ca^2+^) imaging, and protein secretion analysis. The response to the known chemotherapeutic agent temozolomide (TMZ) was used for model validation. Drug response analysis included live cell imaging, ICC staining, and Ca^2+^ imaging.

#### 2.2.2 Neuronal Differentiation

Cortical neurons were differentiated from the two in‐house generated hiPSC lines (UTA.04511.WTs and UTA.10902.EURCCs) and the RFP‐tagged commercial hiPSC line (TUBA1B WTC) according to a previously published protocol [26]. The cell lines are referred to in numerical order as suffixes 1 (UTA.04511.WTs), 2 (TUBA1B WTC) and 3 (UTA.10902.EURCCs) in the established cocultures and neuronal control cultures.

Cortical neurons were utilized on Days 32–33 of the differentiation process for the experiments. Medium used for the cortical neurons was a neuronal maturation medium (NMM), which included 0.5 × Dubecco's Modified Eagle Medium (DMEM)/F‐12 with GlutaMAX, 0.5 × Neurobasal, 0.5% N2 supplement, 1% B27 supplement with retinoic acid, 0.5 mM GlutaMAX‐I Supplement, 0.5% non‐essential amino acids, 50 μM 2‐mercaptoethanol (all from Thermo Fisher Scientific, Waltham, Massachusetts, USA), 2.5 μg/mL insulin (Sigma‐Aldrich, Saint Louis, Missouri, USA), 0.1% penicillin/streptomycin (Thermo Fisher Scientific, Waltham, Massachusetts, USA), 20 ng/mL brain‐derived neurotrophic factor (BDNF, R&D Systems, Minneapolis, Minnesota, USA), 10 ng/mL glial‐derived neurotropic factor (GDNF, R&D Systems, Minneapolis, Minnesota, USA), 500 μM dibuturyl‐cyclic AMP (Sigma‐Aldrich, Saint Louis, Missouri USA), and 200 μM ascorbic acid (Sigma‐Aldrich, Saint Louis, Missouri, USA).

#### 2.2.3 2D Culture of LN229/GFP Cells

LN229/GFP cells were cultured in two‐dimensional (2D) format for morphological characterization and the initial TMZ dose–response studies. LN229/GFP cells were plated on 48‐well plates (Thermo Fisher Scientific, Waltham, Massachusetts, USA) at a density of 11 000 cells per well. The cells were cultured in LN229‐medium for 7 days at 37°C and 5% CO_2_. For the drug dose–response experiments, TMZ (Thermo Fisher Scientific, Waltham, Massachusetts, USA) was diluted in dimethyl sulfoxide (DMSO, Sigma‐Aldrich, Saint Louis, Missouri, USA) and mixed into LN229‐medium at a final concentrations of 0, 50, 100, 200, 400, and 600 μM and applied to the cells 3 days after plating for 7 days in total.

#### 2.2.4 GB Spheroid Formation

LN229/GFP cells were cultured on ultra‐low attachment (ULA) 6‐well plates (Corning Incorporated, Somerville, Massachusetts, USA) to induce spontaneous spheroid formation. A total of 3 mL of LN229‐medium at a concentration of 50 000 cells/mL was used per well. Spheroid cultures were maintained at 37°C and 5% CO_2_. After 3–4 days, the formed spheroids were dissociated to prevent them from becoming too large and developing a necrotic core. The spheroids were collected into a Falcon tube and washed once with 2 mL of phosphate buffer saline (PBS, EuroClone, Pero, Italy). Next, 2 mL of TrypLE (Thermo Fisher Scientific, Waltham, Massachusetts, USA) was added to the tube, and the spheroids were incubated for 5 min at 37°C. After incubation, the suspension was pipetted 70–100 times to break up the spheroids. Then, 5 mL of LN229‐medium was added to inactivate the TrypLE, after which the mixture was centrifuged for 5 min at 200 × g. The cells were resuspended in 1 mL of fresh LN229‐medium and plated on ULA 6‐well plates at a density of 50 000 cells/mL to generate spheroids. Spheroids for 3D experiments were collected on Day 3 or 4 after replating.

#### 2.2.5 Preparation of 3D Neuronal Cultures

Neuronal 3D cultures were prepared using collagen I (Collagen I, Rat Tail, 3 mg/mL, Thermo Fisher Scientific, Waltham, Massachusetts, USA) as previously described [27] at a final concentration of 1 mg/mL. First, 10 × PBS, 1 M sodium hydroxide (NaOH) and distilled water (dH_2_O) were mixed on ice, after which 3 mg/mL collagen I stock solution was added. Next, cortical neurons were embedded in the gel solution at a density of 2.5 × 10^6^ cells/mL. After proper mixing, 200 μL of the cell–hydrogel mixture was pipetted into the wells of 24‐well plates with glass bottoms 13 mm in diameter (MatTek Corporation, Ashland, Massachusetts, USA). The hydrogels were allowed to gel for 30–60 min at 37°C and 5% CO_2_, after which 1 mL of NMM supplemented with 2 μL/mL Rho kinase inhibitor (ROCK, STEMCELL Technologies, Vancouver, Canada) was carefully pipetted on top of each hydrogel. From the next day onward, the medium was changed to NMM without ROCK. 3D neuronal cultures were kept for 7 days in monoculture at 37°C and 5% CO_2_. The NMM was changed every 2 to 3 days.

#### 2.2.6 Formation of the 3D Neuron–GB Model

After 7 days of culturing the neurons in collagen I hydrogels, LN229/GFP GB cell spheroids were added. First, the medium was removed and LN229/GFP spheroids with a diameter of 150–300 μm were selected and pipetted gently on top of the 3D neuronal culture, with one spheroid per sample. Fresh coculture medium containing 1:1 LN229‐medium and NMM was added on top of each sample. The spheroids slowly sank in the middle of the 3D neuronal culture. Cocultures were maintained at 37°C and 5% CO_2_ for 2–4 weeks. The medium was changed every 2 to 3 days.

For control samples containing only GB spheroids (hereafter referred to as GB controls), plain collagen I hydrogel samples were prepared as described in subchapter 3.3 without adding neurons to the hydrogel solution. For control samples containing only neurons (hereafter referred to as neuron controls), samples were prepared as for the cocultures without the addition of GB spheroids on Day 7. Otherwise, the control samples were subjected to the same procedures as the coculture samples.

#### 2.2.7 Immunocytochemical (ICC) Staining of 2D Samples

On Day 7, 2D samples on 48‐well plates were first washed once with PBS for 5 min and then fixed with 4% paraformaldehyde (PFA, Acros Organics, Antwerpen, Belgium) in PBS for 15 min. Fixed samples were subjected to ICC staining according to a previously published protocol [28]. Briefly, the samples were blocked for 45 min with 10% normal donkey serum (NDS, Millipore, Burlington, Massachusetts, USA), 0.1% Triton X‐100 (Sigma‐Aldrich, Saint Louis, Missouri, USA), and 1% bovine serum albumin (BSA, Sigma‐Aldrich, Saint Louis, Missouri, USA) in PBS. Next, samples were washed twice with 1% NDS, 0.1% Triton X‐100, and 1% BSA in PBS. The samples were then incubated overnight at +4°C with the following primary antibodies diluted in PBS with 1% NDS, 1% BSA, and 0.1% Triton X‐100: anti‐β‐tubulin III (βtub_III_, rabbit, 1:500, A01627, GenScript, Piscataway, New Jersey, USA), anti‐glial fibrillary acidic protein (GFAP, chicken, 1:4000, ab4674, Abcam, Cambridge, UK), anti‐S100 calcium binding protein B (S100β, 1:500, mouse, S2532, Sigma‐Aldrich, Saint Louis, Missouri, USA), and anti‐nestin (mouse, 1:1000, MAB5326, Millipore, Burlington, Massachusetts, USA). The next day, the samples were washed twice for 5 min with 1% BSA in PBS. Thereafter, the samples were incubated with the following Alexa Fluor‐labeled secondary antibodies diluted in PBS with 1% BSA: donkey anti‐mouse 647 (A31571, 1:400), donkey anti‐rabbit 568 (A10042, 1:400), and goat anti‐chicken 647 (A21449, 1:400, all from Thermo Fisher Scientific, Waltham, Massachusetts, USA) for 1 h at room temperature, followed by two washes with PBS and one with phosphate buffer. The samples were then mounted with Prolong gold (Thermo Fisher Scientific, Waltham, Massachusetts, USA) containing 4',6‐diamidino‐2‐phenylindole (DAPI) for nuclear staining and covered with 9 mm coverslips.

#### 2.2.8 ICC Staining of 3D Samples

3D samples were fixed after 2–4 weeks of coculturing as previously described [27, 29]. The samples were washed once with PBS for 5 min and then fixed with 4% PFA in PBS for 1 h at room temperature. After the samples were washed twice with PBS for 10 min, they were blocked for 1 h with blocking solution containing 10% NDS, 0.1% Triton X‐100, and 1% BSA in PBS. Next, samples were washed once with 1% NDS, 0.1% Triton X‐100, and 1% BSA in PBS for 5 min. The samples were then incubated for 3 days on a shaker at +4°C with the following primary antibodies diluted in PBS with 1% NDS, 1% BSA, and 0.1% Triton X‐100: anti‐βtub_III_ (rabbit, 1:250, A01627 or chicken, 1:100, AB41489, Abcam, Cambridge, UK), anti‐microtubule associated protein 2 (MAP‐2, rabbit, 1:200, AB5622, Millipore, Burlington, Massachusetts, USA or chicken, 1:2000, NB300‐213, Novus Biologicals, Centennial, Colorado, USA), anti‐S100β (1:250, mouse, S2532), anti‐postsynaptic density protein 95 (PSD95, mouse, 1:50, AB2723, Abcam, Cambridge, UK), and anti‐synapsin‐1 (rabbit, 1:500, 574777, Millipore, Burlington, Massachusetts, USA). Then, the samples were washed with 1% BSA in PBS 5 times: 1 × quick wash, 1 × 7 h wash, 1 × overnight wash, 1 × 7 h wash, and 1 × overnight wash. The samples were then incubated with the following Alexa Fluor‐labeled secondary antibodies diluted in PBS with 1% BSA: donkey anti‐mouse 647 (A31571, 1:200) or goat anti‐chicken 647 (A21449, 1:200), donkey anti‐rabbit 568 (A10042, 1:400) or goat anti‐chicken 568 (A11041, 1:400, Thermo Fisher Scientific, Waltham, Massachusetts, USA), goat anti‐mouse 405 (A31553, 1:125, Thermo Fisher Scientific, Waltham, Massachusetts, USA) and DAPI (D9542, 1:5000, Sigma‐Aldrich, Saint Louis, Missouri, USA) overnight at +4°C on a shaker, followed by 5 washes with PBS: 1 × quick wash, 1 × 1 h wash, 1 × 3 h wash, and 2 × 15 min washes. The samples were left in fresh PBS and stored at +4°C in the dark.

#### 2.2.9 Image Acquisition and Image Analysis

The samples were imaged with a set of several microscopes. During cell culture, phase contrast images were acquired with an ECLIPSE Ti‐S (Nikon, Minato, Japan) or Axio Vert.A1 (Zeiss, Oberkochen, Germany) microscope. Fixed and stained 2D samples were imaged with an IX51 fluorescence microscope (Olympus, Tokyo, Japan) and fixed and stained 3D samples were imaged with a DMi8 widefield fluorescence microscope (Leica, Wetzlar, Germany) and LSM 780 or 880 laser scanning confocal microscopes (Zeiss, Oberkochen, Germany). The deconvolution of fluorescence images was performed with Huygens Essential software (Scientific Volume Imaging, Hilversum, The Netherlands). The acquired images were processed with ImageJ software [30].

2D GB cell coverage under 0–600 μM TMZ treatment was quantified in ImageJ after 7 days of treatment. The GFP fluorescence images taken with the DMi8 widefield fluorescence microscope using the 5× objective were imported and converted to 8‐bit, followed by contrast enhancement using the Auto function. To segment cells from the background, a Mean thresholding method was applied, generating binary masks from which the cell‐covered area was measured. These measurements were used to compare total cell areas across the different TMZ treatment concentrations.

GB invasion rates were determined as follows: First, GBM spheroid diameters were determined from GFP fluorescence images taken on Day 8 (1 day after spheroid plating) and Days 14, 21, or 28. From the images, each spheroid was measured from three different points so that the final spheroid diameters were the mean values. The final diameters were divided by 2 to obtain the spheroid radius. Finally, a spheroid's radius at an earlier time point was subtracted from the radius at a later time point to obtain the change in spheroid size, which was then divided by the days past between the time points, resulting in an average invasion rate per day.

#### 2.2.10 Ca^2+^ Imaging and Analysis

Live cell Ca^2+^ imaging was performed with 3D hydrogel samples at Days 21–28 of cell culture following a recently described protocol [31]. Samples for Ca^2+^ imaging were washed 3 times with extracellular solution (ECS, 137 mM NaCl, 5 mM KCl, 0.44 mM KH_2_PO_4_, 20 mM HEPES, 4.2 mM NaHCO_3_, 5 mM D‐glucose, 2 mM CaCl_2_, 1.2 mM MgCl_2_ and 1 mM Na‐pyruvate), followed by 1 × quick wash and 2 × 5 min washes. The samples were then treated with 5 μM Rhod‐4 AM (AAT Bioquest, Pleasanton, California, USA) in the ECS and incubated for 1 h at 37°C to load the cells with the Rhod‐4 AM Ca^2+^ indicator. The samples were washed once quickly and once for 30 min with ECS at 37°C. Approximately 2‐min‐long time‐lapse image series were taken with a DMi8 fluorescence microscope from coculture, neuron control, and GB control samples to measure spontaneous Ca^2+^ activity. Additionally, images of GFP‐tagged GB cells were acquired to differentiate between neurons and GB cells in the coculture samples as follows: The GFP images were converted into binary images using ImageJ, employing a threshold set to their mean intensity value. The coculture time‐lapse series were then multiplied with binary masks to obtain time‐lapse series of the cocultures' GB sides and neuron sides separately for analysis (Figure S5a).

For analyzing Ca^2+^ imaging time lapse images, first, ImageJ was used with an additional plugin, Time Series Analyzer V3 (TSA) (https://imagej.nih.gov/ij/plugins/time‐series.html). All active cell areas in each imaged region were semiautomatically detected by first taking the maximum and minimum intensities of each pixel across the time lapse and subtracting the minimum from the maximum. The resulting image was filtered with a low‐pass filter using the default configuration in ImageJ. Then, a thresholding value of 160–200 was used to filter out background noise, and subsequently, the objects were smoothed with a median filter at a radius of 5 pixels. Next, regions of interest (ROIs) representing detected active cells were placed at intensity locations with ImageJ's Analyze Particles function, including further filtering of ROIs outside the range of 50–200 square pixels to prevent misdetection of particles other than cells. An additional ROI was manually added to a location without any biological fluorescence signal to define the background intensity of the images. Finally, the Get Average function of the TSA was applied to derive activity data from each ROI of the whole timelapse stack.

The Ca^2+^ activity data obtained from ImageJ were further processed using a custom code with MATLAB R2023 v.9.14.0 (The MathWorks Inc., Natick, Massachusetts, USA). First, the background signal was subtracted from the raw data, after which fast frequency noise was removed by filtering the signal with a 10‐point moving average filter. Next, signal values were calculated as

$$∆F/F_{0},$$

where ΔF is the signal intensity change and $F_{0}$ is the base fluorescence level defined as the mean of the lowest 20% of the signal. After additional normalization with linear regression, signal peaks were searched with the *findpeaks* function in MATLAB using a peak prominence threshold of 0.07 units, and only peaks with absolute heights above the mean value of the signal were counted. Finally, parameters including the peak amplitude, prominence, peak duration, rising and descending slope angles of the peaks, peak‐to‐peak distance, and frequency were determined. The MATLAB code for the analysis is available at https://github.com/VVuolanto/Calcium‐Imaging.

The percentage of active neuronal area was calculated by dividing the area of active neurons by the total area of neurons. The area values were derived from neuron controls and from cropped coculture image series from which regions containing GB cells were excluded (Figure S5b). The percentage of active neurons in each analyzed sample region was calculated as

$$\text{Active neuronal area}\;\left(\%\right)=\frac{A\left(\text{active}\right)}{A\left(\text{total}\right)}\times 100,$$

where $A\left(\text{active}\right)$ is the pixel area of all active cells in the region and $A\left(\text{total}\right)$ is the pixel area of all stained (active and nonactive) cells in the region. $A\left(\text{active}\right)$ was obtained by summing the pixel counts of the detected active ROIs found in each time‐lapse region. The pixel area of all stained cells, that is, $A\left(\text{total}\right)$, was obtained via ImageJ's Analyze Particles function, with manual thresholding to include all stained cell areas. ROIs smaller than 50 square pixels were discarded while calculating the total neuronal area to prevent misdetection of debris.

#### 2.2.11 Protein Secretion Analysis

A Proteome Profiler Human XL Cytokine Array Kit (R&D Systems, Minneapolis, Minnesota, USA) was used to analyze protein secretion from the cells according to the manufacturer's instructions. Briefly, conditioned medium was collected from cocultures, neuron controls, and GB controls on Day 19. The array membranes were imaged with a ChemiDoc XRS+ system with Image Lab software (Bio‐Rad, Herculer, California, USA). Pixel densities from the imaged membrane spots were defined with OptimEyes/HLImage++ QuickSpot software (Ideal Eyes Systems, New York, USA). The graphs were generated with GraphPad Prism 10.0.1 (GraphPad Software, La Jolla, California, USA).

#### 2.2.12 3D Drug Testing Assay

TMZ was applied to 3D hydrogel cultures to assess the drug response of the cells. TMZ was diluted in DMSO and added to the cell culture medium at a concentration of 400 μM. Drug application was started on Day 14, 7 days after GB spheroid plating. The samples were treated with TMZ for 7, 14 days, or for 7 days followed by 7 days without TMZ. The control groups were treated with only medium or with medium supplemented with 1% DMSO (vehicle control). Additionally, 4000 μM TMZ or correspondingly medium supplemented with 10% DMSO for the vehicle control was applied to a few samples from Day 14 to Day 21 to evaluate the effect of higher drug concentration.

#### 2.2.13 Quantification and Statistical Analysis

Statistical analyses were performed, and the graphs were plotted using GraphPad Prism version 10.0.1. Exact values of *n* can be found in figure legends and Tables S2 and S3. The normality of the data was checked using Shapiro–Wilk's normality test (*n* < 50) or with Kolmogorov–Smirnov's normality test (*n* ≥ 50). Due to the non‐Gaussian distribution, nonparametric tests were used for calculating the significant differences. The statistical significance of the difference in the invasion rate of GB spheroids in cocultures and in the GB control group was calculated with a nonparametric Kruskal–Wallis test with Dunn's correction for multiple comparisons. The nonparametric Mann–Whitney *U*‐test was used to calculate the significant differences in average spheroid invasion per day between the medium‐treated and TMZ‐treated groups, in terms of the intracellular Ca^2+^ dynamics between mono‐ and cocultured cells, in terms of the percentage of the active neuronal area between the medium‐treated and TMZ‐treated neuron cell line 3 samples, and in terms of the intracellular Ca^2+^ dynamics between the TMZ‐treated and control cocultures. The statistical significance of the difference in the 2D GB cell area coverage in 0–600 μM TMZ was calculated with a nonparametric Kruskal–Wallis test with Dunn's correction for multiple comparisons. A significance level of < 0.05 was used. Statistical significance is denoted as * < 0.05, ** < 0.01, *** < 0.001, and **** < 0.0001.

## 3 Results

### 3.1 Establishment of Long‐Term 3D Neuron‐GB Model

For this work, the GB cell line LN229 was chosen because it is a line commonly used in in vitro studies [21, 25] and it is available with a green fluorescent protein (GFP) fluorescence tag. The LN229/GFP cells used were characterized before they were cocultured. Under adherent 2D culture conditions, LN229/GFP cells exhibited the characteristic morphology and typical expression of glial and neuronal markers (Figure S1a), whereas under nonadherent conditions, they started to form spheroids within a couple of days (Figure S1b).

To establish a 3D neuron–GB coculture model, collagen I was used to form the 3D culture environment. First, hiPSC‐derived cortical neurons were embedded in collagen I hydrogel. The neurons were allowed to grow and form networks for 7 days, after which the GB spheroids (3–4 days old) were embedded in the samples. Cocultures were maintained for 2–4 weeks; thus, the longest experiment was 35 days in total. A schematic overview of the model setup is shown in Figure 1a.

The developed coculture model was both viable and reproducible. ICC staining of samples after 2 weeks of coculture revealed that neurons formed consistent neuronal networks expressing microtubule‐associated protein 2 (MAP‐2) and beta tubulin III (βtub_III_) (Figure 1b). This was confirmed with neurons derived from three different hiPSC lines, which are marked here as suffixes 1 (UTA.04511.WTs), 2 (TUBA1B WTC) and 3 (UTA.10902.EURCCs) when discussing cocultures and neuronal control cultures. Some cells also expressed S100 calcium‐binding protein β (S100β), indicating differentiation toward the astrocyte lineage. Furthermore, after invasion into the surrounding neuronal networks, the LN229/GFP spheroids were viable (Figure 1b) which was confirmed via 3D visualization (Video S1). Steady spheroid growth during the coculture period was evident (Figure 1c) and the quantified spheroid invasion rates were 106.4 ± 19.4, 134.4 ± 10.4, 119.5 ± 13.5, and 121.5 ± 16.8 μm/day for cocultures 1, 2, 3, and GB monocultures, respectively. The spheroids in coculture 1 exhibited a lower invasion rate than those in coculture 2 (*p* = 0.0005) but overall the spheroids exhibited similar invasion rates under all conditions (Figure 1d).

The developed model remained intact after 4 weeks of coculture, with the cells maintaining their characteristic morphologies (Figure S1c). However, at the 4‐week coculture time point, the LN229/GFP spheroids overgrew and covered most of the sample area. In the overgrown samples, the GBM cells also grew along the bottom of the culture dish (data not shown), which is not optimal for visualization and analysis.

### 3.2 Neurons and GB Cells Show Physical Interactions in 3D Cocultures

**FIGURE 2:**
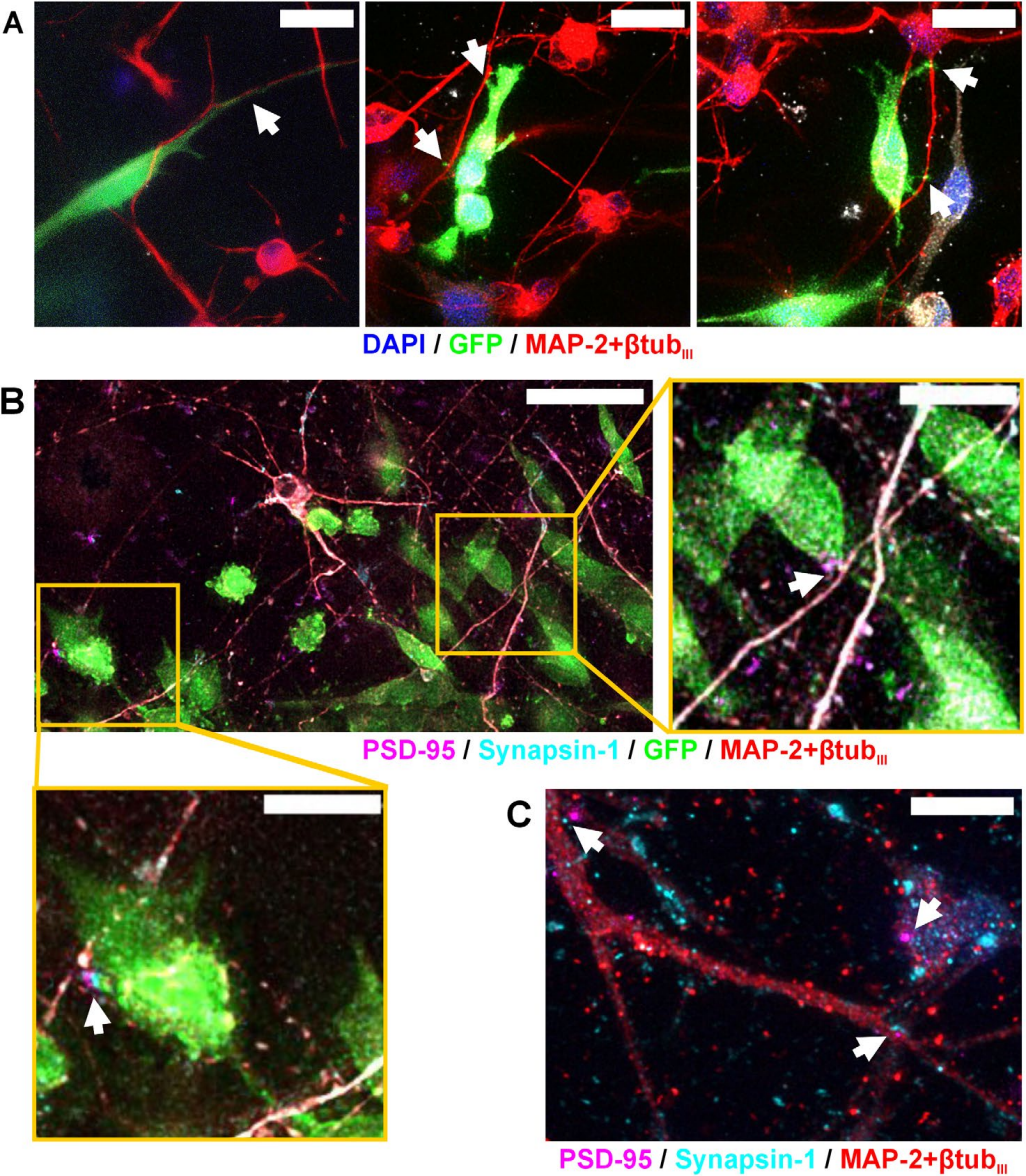
Physical interactions between neurons and GB cells in 3D cocultures. (a) Physical contact between neurons (MAP‐2 + ßtub_III_) and GB cells (labeled with GFP) was observed at the border area of the GB spheroids on Day 21. The white arrows indicate points where the GB cells grew in proximity to the neurons and were aligned next to the neurites. Cell nuclei were stained with DAPI. The scale bar is 25 μm. (b) The presence of synaptic markers (synapsin‐1 and PSD‐95) was identified in cocultures. Zoomed images show that some of the synapses were colocalized in the neuron–GB interface (indicated by arrows). The scale bar is 50 μm, in the zoomed‐in parts it is 20 μm. (c) Neurons in neuron controls expressed pre‐ and postsynaptic markers (synapsin‐1 and PSD‐95) in the collagen I environment. The scale bar is 10 μm. See also Figure S2.

Closer examination of the neuron–GB interface via confocal microscopy revealed the close proximity of the two cell types, suggesting physical cell–cell interactions. The images revealed GB cell growth toward neurons and along neurites (Figure 2a). Figure 2c shows that neuron monocultures in the collagen I hydrogel expressed pre‐ and postsynaptic markers synapsin‐1 and postsynaptic density protein 95 (PSD‐95). Similarly, in cocultures, the colocalization of synaptic markers at neuron–GB contact points was observed, indicating the presence of synaptic contacts between them (Figure 2b). Furthermore, neuronal networks appeared similar in both cocultures and neuron controls (Figure S2), suggesting that their integrity was not disrupted by GB cells.

### 3.3 Protein Secretion Analysis Indicates Tumor‐Modulating Paracrine Interactions in 3D Coculture

**FIGURE 3:**
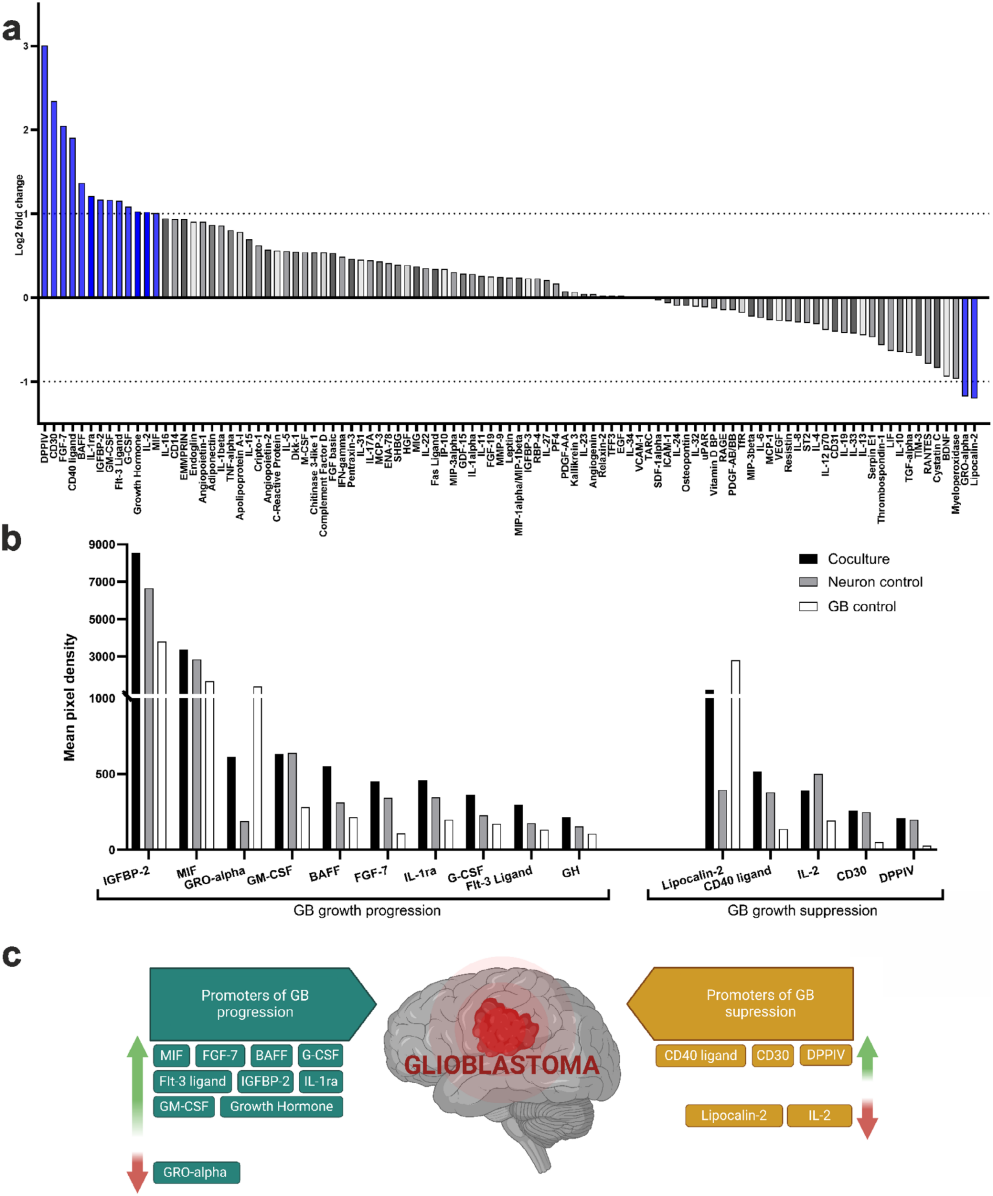
Protein secretion analysis indicates tumor‐modulating paracrine interactions in 3D coculture. (a) From all 105 analytes, proteins were selected for further examination based on the relative Log_2_ fold change values between cocultures and GB controls. Proteins that are presented as negative fold change values were secreted more in GB controls and proteins with positive fold change were secreted more in cocultures. Threshold (dotted line) was set to log_2_ fold change ±1 to select the proteins which secretion had changed substantially. (b) Quantified mean pixel density values from protein secretion array membranes depicting the secretion profiles of the selected proteins in coculture, neuron control and GB control conditions. The proteins include insulin‐like growth factor binding protein 2 (IGFBP‐2, encoded by the IGFBP2 gene), macrophage migration inhibitory factor (MIF, MIF gene), GRO‐alpha (CXCL1 gene), granulocyte‐macrophage colony‐stimulating factor (GM‐CSF, CSF2 gene), B‐cell activating factor (BAFF, TNFSF13B gene), fibroblast growth factor 7 (FGF‐7, FGF7 gene), interleukin (IL)‐1 receptor antagonist (IL‐1ra, IL1RN gene), granulocyte colony‐stimulating factor (G‐CSF, CSF3 gene), FMS‐like tyrosine kinase 3 ligand (Flt‐3 ligand, FLT3LG gene), growth hormone (GH, GH1/2 gene), lipocalin‐2 (LCN2 gene), CD40 ligand (CD40LG gene), IL‐2 (IL2 gene), CD30 (TNFRSF8 gene), and dipeptidyl peptidase 4 (DPPIV, DPP4 gene). (c) The most substantially altered protein secretion levels were grouped into promoters and suppressors of GB growth. Green and red arrows indicate increased and decreased secretion levels, respectively, in the coculture compared to the GB control conditions. Created in BioRender. Isosaari, L. (2025) https://BioRender.com/i53n437.

Protein secretion analysis was performed as a descriptive approach, similar to previous studies [32, 33], to detect possible protein level changes between cocultures and controls. In total, 105 analytes (Table S1) were studied from the cell culture media on Day 19 (Figure S3b). The analysis revealed bidirectional paracrine signaling in coculture conditions, indicated by differences in relative protein secretion compared to that of neuron or GB controls. Proteins were selected for further examination based on the relative changes between coculture and GB control. The threshold for log_2_‐fold change was set to ±1 to select the most substantial changes (Figure 3a).

Differentially secreted proteins were grouped based on their reported functions in GB pathology (Figure 3a). In general, various proteins that either support or suppress GB progression were differentially secreted in the coculture condition compared to the GB control (Figure 3b). For example, coculture conditions increased the secretion of the potent tumorigenic proteins IGFBP‐2, MIF, BAFF, FGF‐7, IL‐1ra, G‐CSF, Flt‐3 ligand, and growth hormone. Coculture conditions also increased the expression of potential GB growth suppressors, such as the CD40 ligand, CD30, DPPIV, and IL‐2, compared to those in the GB control group, while some of these factors were secreted to a similar extent in the neuron control group. Thus, the secretion of some detected suppressive factors can be explained by natural neuronal secretion (Figure 3b). Compared with protein secretion in the neuron control group, only IL‐2 secretion was decreased in the coculture group. Compared to those of the GB control, the coculture conditions had lower levels of GRO‐alpha and lipocalin‐2. Thus, the protein secretion analysis results indicate paracrine neuron–GB crosstalk in the model.

### 3.4 Functional Connectivity in Neuron–GB Cocultures

**FIGURE 4:**
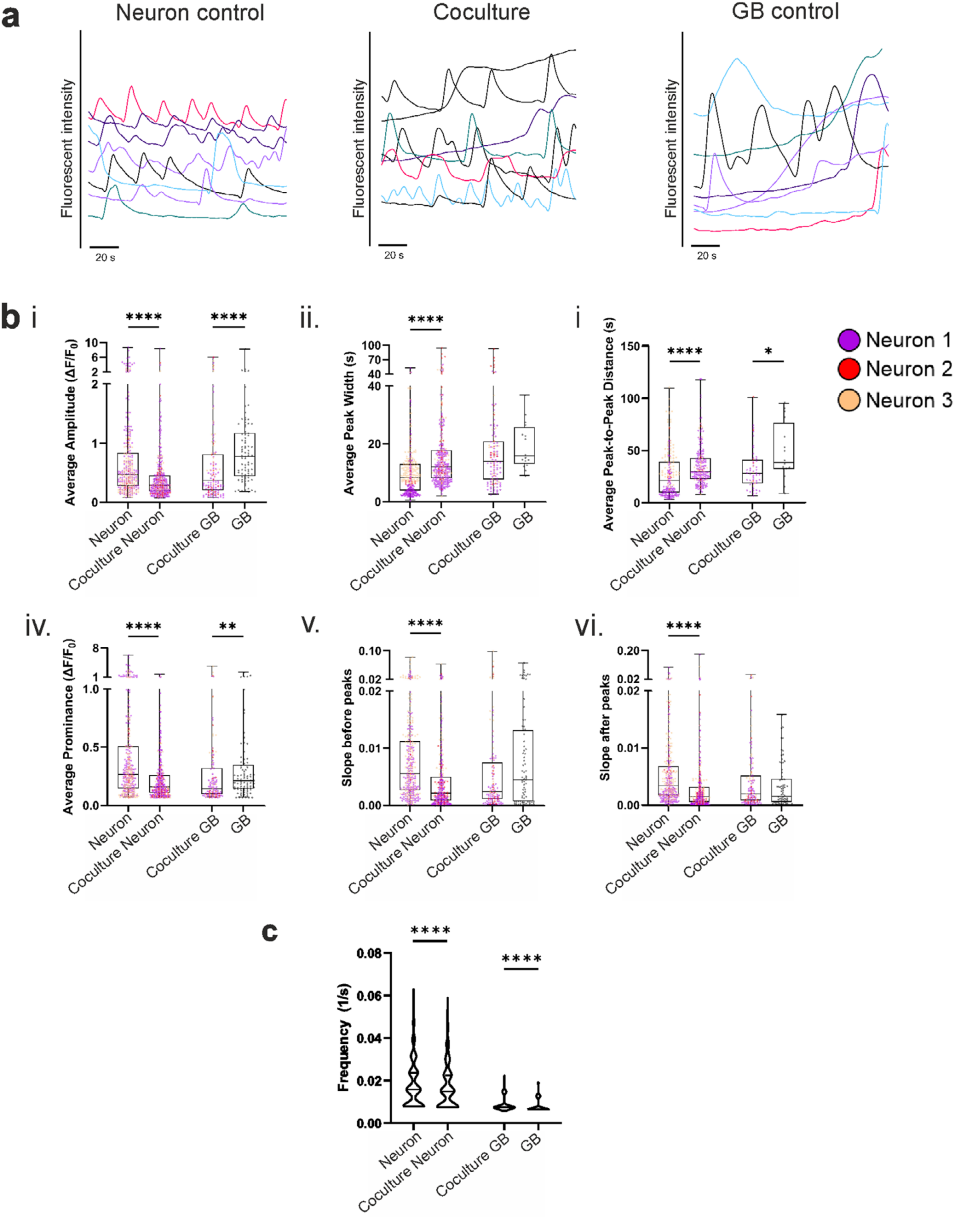
Functional connectivity in neuron–GB cocultures. (a) Representative Ca^2+^ oscillations detected in neuron controls, cocultures, and GB controls. Neurons exhibited faster spiking compared with GB waves that were overall slower. In cocultures, both kinds of oscillations were detected. The activity of all sample types confirmed that the model environment supported cellular functionality. (b) Ca^2+^ parameters (i) average amplitude, (ii) average peak width, (iii) average peak‐to‐peak distance, (iv) average prominence, (v) slope before peaks, and (vi) slope after peaks for controls and cocultured neurons and GB cells. (c) Ca^2+^ oscillation frequencies of controls and cocultured neurons and GB cells. The sample size for each group with each Ca^2+^ parameter is shown in Table S2. Statistical comparisons were performed with the Mann–Whitney *U* test. Significance is indicated by * < 0.05, ** < 0.01, and **** < 0.0001. See also Videos S2 and S3.

In addition to physical and paracrine interactions, functional interactions were assessed in the established model. The functionality of the cells was studied by Ca^2+^ imaging after 2 weeks of coculture. The results showed that both neurons and GB cells were functionally active. The neuronal activity was heterogenous with partially synchronous oscillations and individually active cells. Representative spontaneous Ca^2+^ signals are presented in Figure 4a as well as in Videos S2 and S3. Neurons expressed faster spike‐like spontaneous activity whereas Ca^2+^ oscillations obtained from GB cells were mainly broader and slower. Both fast and slow activity were measured in the coculture samples, indicating that both cell types were active in the cocultures. Moreover, various Ca^2+^ activity parameters for both cell types were determined from the obtained Ca^2+^ oscillations under control and coculture conditions (Figure 4b). Functional differences between cocultured and monocultured cells, both neurons and GB cells, were detected according to the analyzed parameters. Interestingly, most of the neuronal calcium oscillation parameters were significantly altered when the neurons were cocultured with GB (amplitude, prominence, peak‐to‐peak distance, and frequency, *p*‐values < 0.0001). In contrast, the parameters of GB activity did not change as drastically, although significant changes were detected in amplitude, peak‐to‐peak distance, prominence, and frequency (*p*‐values < 0.0001, < 0.05, < 0.01 and < 0.0001, respectively). When comparing the cocultures and monocultures, the Ca^2+^ signal parameters of cocultured cells, both neurons and GB, reached similar levels except for the signal frequency. Thus, the waveforms of both the neuron and GB signals shifted in the cocultures. These findings underscore the effects of cellular interactions on the functionality of both neurons and GB cells.

### 3.5 TMZ Treatment Affects GB Spheroid Growth

**FIGURE 5:**
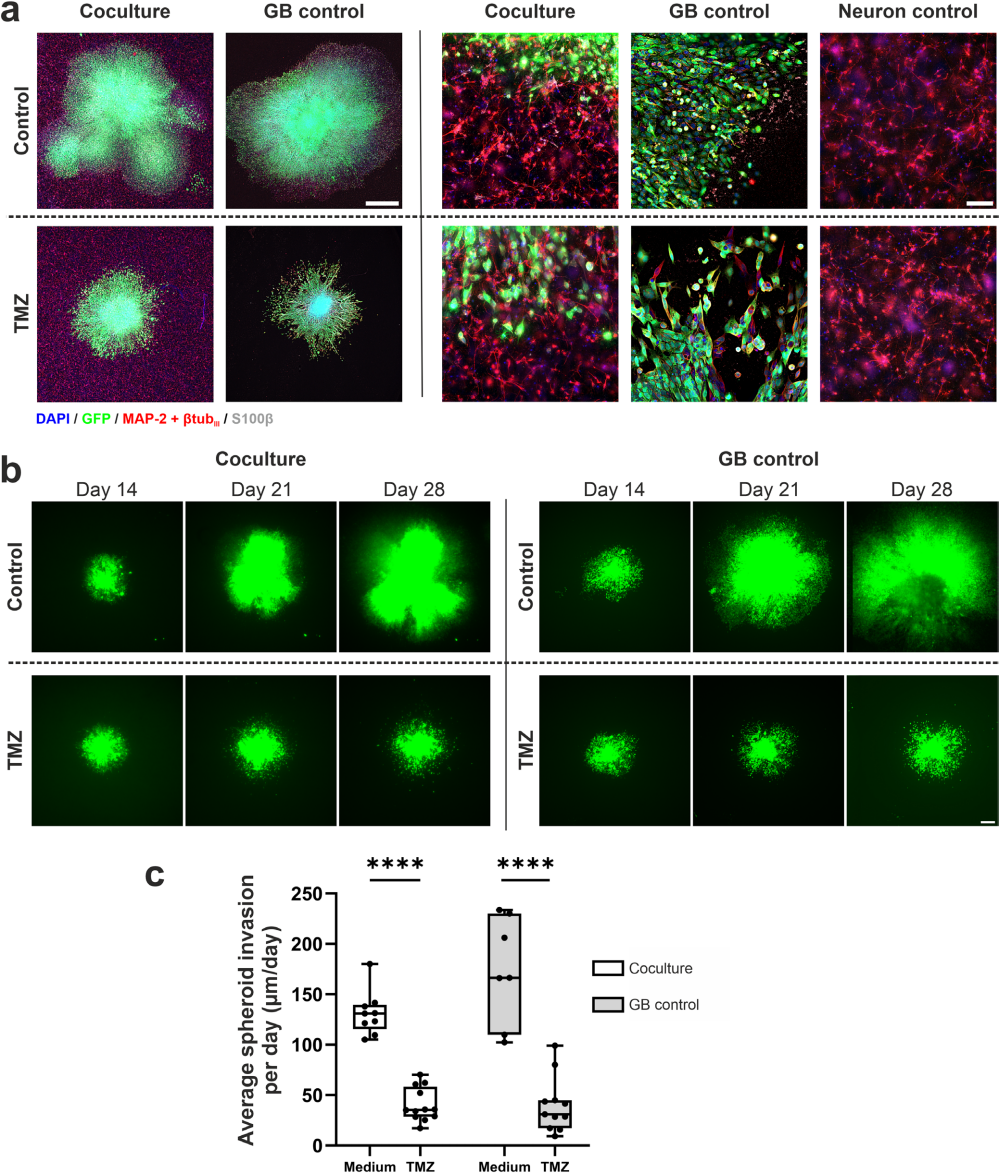
TMZ treatment affects GB spheroid growth. (a) Images of ICC‐stained samples showing that GB spheroids (labeled with GFP) remained smaller in 400 μM TMZ‐treated samples than in vehicle controls (1% DMSO). Neuronal networks (MAP‐2 + βtub_III_) did not show any visual damage under these conditions. Cell nuclei were stained with DAPI. The scale bar on the left is 1000 μm, and the scale bar on the right is 100 μm. (b) Invasion of GB spheroids was followed during TMZ treatment. TMZ application started after one week of coculture clearly decreased and nearly stopped GB spheroid invasion during the next 2 weeks in both the coculture and GB control conditions, while the nontreated samples continued to expand. The scale bar is 500 μm. (c) Quantification of GB spheroid invasion rates during the first week of TMZ treatment (from Day 14 to Day 21) revealed a significant decrease in TMZ‐treated samples. The medium control and vehicle control groups were not significantly different. The Mann–Whitney *U* test was performed to compare the differences between the coculture medium control (*n* = 9) and TMZ‐treated cocultures (*n* = 12) as well as between the GB medium control (*n* = 7) and TMZ‐treated GB cultures (*n* = 11). Significance is indicated by **** < 0.0001. See also Figure S4.

TMZ was used to study the drug response in the developed model. First, we performed 2D dose–response studies with the LN229/GFP cell line (Figure S4a–c). With increasing TMZ concentrations from 50 to 100 μM, GB cells were visibly harmed by the drug, and a more drastic effect was observed from 400 μM onward, which is in line with previous studies on LN229 cells [34]. TMZ responses in 2D do not directly reflect the 3D situation, emphasizing that drug responses in 3D cultures can differ significantly from those observed in 2D monolayers [35]. Thus, the 400 μM TMZ concentration used in experiments was chosen based on both the 2D test and the literature on preclinical 3D models [36, 37]. The 3D neuron–GB coculture model exhibited shrinkage of the tumor mass after 7 days of TMZ treatment (Figure 5a). In the medium control samples, the GB spheroids continued to grow constantly, while TMZ treatment disturbed their growth (Figure 5b). Quantification of the invasion rate revealed that TMZ significantly decreased or even stopped LN229/GFP spheroid invasion (Figure 5c). Results from the vehicle controls containing 1% dimethyl sulfoxide (DMSO) in culture media did not significantly differ from medium controls (data not shown). Some samples were maintained in normal medium conditions after 7 days of TMZ treatment to follow changes in spheroid growth, as it has been observed that GB spheroids can start regrowing within several weeks despite initial TMZ treatment [38]. It seemed that the GB spheroid parts that survived the first 7 days of TMZ treatment were able to resume expansion after TMZ treatment was stopped compared to the samples that were kept in TMZ for another 7 days (Figure S4d). Upon closer visualization of ICC‐stained samples, the neurons were observed to maintain their morphology and network integrity under TMZ conditions (Figure 5a).

### 3.6 Neuronal Functionality Is Maintained During TMZ Treatment in 3D Cocultures

**FIGURE 6:**
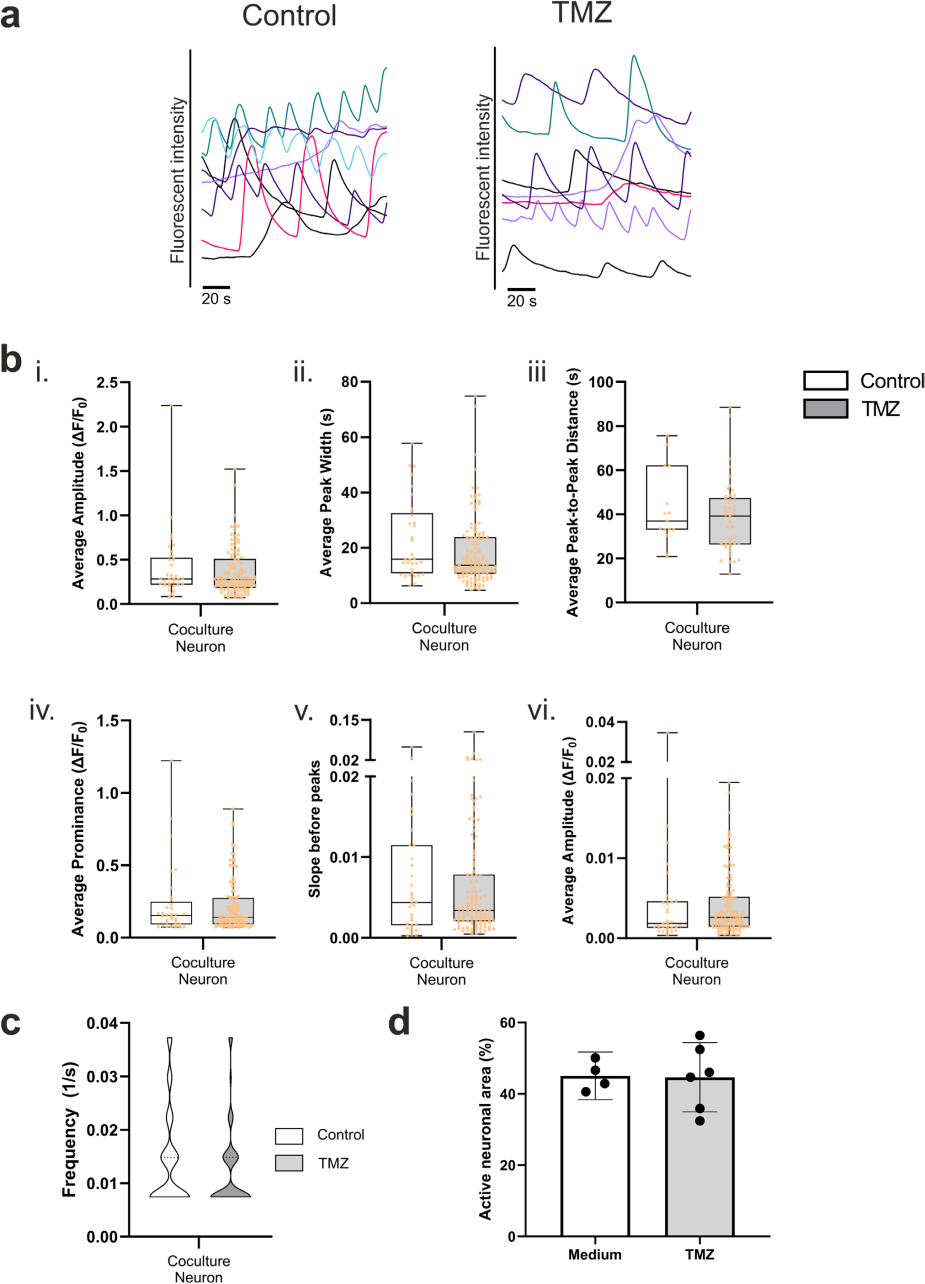
Neuronal functionality is maintained during TMZ treatment in 3D cocultures. (a) Representative Ca^2+^ oscillations detected in TMZ‐treated and untreated cocultures. Spontaneous activity was observed regardless of drug application. (b) Ca^2+^ parameters. (i) Average amplitude, (ii) average peak width, (iii) average peak‐to‐peak distance, (iv) average prominence, (v) slope before peaks, and (vi) slope after peaks for cocultured neurons with and without TMZ treatment. The sample size for each group with each parameter is shown in Table S3. (c) Ca^2+^ oscillation frequency of cocultured neurons with and without TMZ treatment. The sample sizes are shown in Table S3. No significant difference in any Ca^2+^ parameter was found between the control and TMZ‐treated groups. (d) Percentage of active neuronal area in the medium control and TMZ‐treated cocultures. No significant differences were found between the medium control (*n* = 4) and TMZ‐treated (*n* = 6) groups. All of the statistical comparisons were performed with the Mann–Whitney *U* test. See also Figures S5 and S6.

The functional characteristics of the cells were studied in coculture conditions after TMZ treatment via Ca^2+^ imaging. Spontaneous Ca^2+^ oscillations were detected in both neurons and GB cells treated with and without TMZ (Figure 6a). Because drugs can induce adverse changes in neuronal functionality, we were particularly interested in neuronal Ca^2+^ parameters under TMZ‐treated and nontreated coculture conditions. The signals from untreated and TMZ‐treated neurons did not significantly differ from each other (Figure 6b,c). Thus, these results support the safety of 400 μM TMZ treatment on neuronal functionality in vitro.

The studied Ca^2+^ parameters describe the characteristics of the detected Ca^2+^ signals but do not reveal possible changes in the total number of active neurons. Therefore, the percentage of active neuronal area was determined by dividing the area of active neurons by the area of all neurons in an image area to evaluate the overall neuronal activity. The analyzed areas included only neurons close to the GB spheroids (Figure S5b). Treatment with 400 μM TMZ did not significantly change the total neuronal activity in the cocultures compared to that in the untreated cocultures (Figure 6d), further supporting the safety of 400 μM TMZ treatment for neurons. Similar results were obtained under both medium control conditions (Figure 6d) and vehicle control conditions (Figure S6a). However, treatment with 4000 μM TMZ or 10% DMSO visibly disrupted cell morphology and halted spontaneous Ca^2+^ activity in both neurons and GB cells (Figure S6b–d). This indicates that the developed model can be utilized to distinguish safe and hazardous drug concentrations.

## 4 Discussion

Great interest is put into tumor models that incorporate parts of the human TME, with the hope they could unravel pathological processes leading to novel treatment targets. However, as more GB in vitro models are established, determining the most appropriate model for specific research questions is challenging [4]. For instance, protocols for brain organoids [39] can be utilized to establish 3D GB models. Those models seem to best recapitulate the heterogeneity of tumor cells and maintain parental tumor characteristics [40] whereas matrix‐based models might be more suitable for GB invasion studies [20]. One limitation is the lack of effective data acquisition methods for 3D setups, which are becoming increasingly complex [4]. Here, we developed a human‐based in vitro GB model with several physiologically relevant aspects, including a 3D matrix, the presence of neuronal cells, and the relevant organization of GB cells in preexisting neuronal networks, to enable sophisticated analysis of 3D cell interactions and drug responses, also considering cellular functionality, perineural invasion, and host cell safety.

Collagen I hydrogel was selected as the matrix material because it has commonly been used for both 3D central nervous system and GB models [21, 27, 41]. The hydrogel consisted of 1 mg/mL collagen I, which lies in a reported suitable collagen concentration range (0.5–1.5 mg/mL) for studying GB invasion [41]. Our results confirmed that collagen I is suitable for establishing 3D neuron–GB cocultures, as indicated by the morphology and marker expression of both the neuronal networks and LN229/GFP cells in a cell type–specific manner [21, 25]. Moreover, the obtained spheroid invasion rates were consistent with earlier results in which LN229 spheroids invaded 400 μm in collagen I hydrogel within 3 days [42]. Previously, rat‐derived neurons have been shown to affect the invasion of patient‐derived GB cells both in 2D and 3D [20, 43]. A recent study demonstrated that the impact of 2D mouse neuron cocultures on GB proliferation varies depending on the epigenetic profiles of the GBs, and only GBs with high‐neural signature had increased proliferation in cocultures [44]. In our study, the human cortical neurons did not significantly enhance GB invasion compared with GB‐only cultures. The utilized LN229 cells are characterized by rapid proliferation but little invasion compared to other GB cell lines, which have a more invasive nature [21] which can explain their unchanged invasion rate in the presence of human neurons. Moreover, as the LN229 has limitations in representing the heterogeneity of the original GB tumor, in future studies, the established model can be utilized with patient‐derived GB spheroids for a more comprehensive assessment of neuron–GB interactions in the invasion properties.

Neuronal network formation was successful in collagen I as shown previously [27], also in the presence of GB. ICC staining revealed the development of a consistent neuronal network repeatedly throughout several experiments with neurons derived from different hiPSC lines. Visualization of synaptic markers revealed that neurons can form synaptic connections with each other and with GB cells in 3D. Our study extends previous works showing functional synaptic connections between high‐grade glioma cells and neurons in 2D [6, 7] by displaying the cellular connections in the proximity of the two cell types at the GB border zone and colocalized expression of synaptic markers on the cell membranes. To our knowledge, this is the first time that neuron–GB synapses have been investigated in vitro in 3D.

Bidirectional paracrine communication between neurons and glioma cells has been reported to induce tumor progression as well as to promote neuronal hyperexcitability [12, 45]. Here, protein secretion analysis revealed paracrine neuron–GB crosstalk, with a secretion pattern that can support GB growth. For instance, the phosphatidylinositol 3‐kinase (PI3K)/Akt/rapamycin‐sensitive mTOR‐complex (mTOR) pathway is one of the key signaling pathways activated and affected in gliomas, and associated mutations can be found in most GB patients [46]. In our coculture system, the secretion of FGF‐7, IGFBP‐2, and growth hormone, which are known to activate this pathway [47, 48, 49], was elevated. High levels of IGFBP‐2 can be explained by its involvement in neural development, growth, and activity [50], while in GB patients, increased expression of the IGF signaling axis, for example, IGFBP‐2 secretion, has been associated with malignant tumor progression and TMZ resistance [49, 51]. Thus, paracrine neuron–GB interactions can potentially also affect drug responses. Coculture conditions increased the secretion of the tumorigenic proteins BAFF, IL‐1ra, G‐CSF, Flt‐3 ligand, and MIF, which all support tumor proliferation and/or progression [52, 53, 54, 55, 56]. Under neuron control and coculture conditions, the secretion of GM‐CSF, which is known to induce immunosuppression in the TME and support glioma progression [57], was similar. Thus, the secretion of tumor‐supportive factors by neuronal cells was detected in the cultures. Moreover, the level of IL‐2, an antitumor cytokine [58], was decreased in the coculture compared to the neuron control, suggesting that neuron–GB communication can decrease the levels of tumor suppressive factors naturally secreted by neurons and/or astrocytes. The neuronal cultures utilized here also contained minor populations of astrocytes. The secretion pattern in the cocultures did not solely support tumor progression. Compared to the GB control, coculture conditions decreased the secretion of GRO‐alpha, a tumor‐supportive factor [59], and increased the secretion of the antitumor factors CD40 ligand and CD30 [60, 61]. Neuronal monocultures secreted DPPIV, a glioma suppressive factor widely expressed in both neurons and astrocytes [62, 63], at similar levels as cocultures. The levels of some detected suppressive factors can be explained by the natural secretion of neurons and astrocytes.

Electrophysiological functionality is one of the key features that should be measured when modeling pathophysiological processes in the brain. Currently available 3D in vitro models lack comprehensive functional assessments to study neuron–GB interactions. Most of the functional data originate from 2D setups or animal models [6, 7, 8, 9, 44, 45] which have reported the ability of GB cells to disrupt neuronal activity in vivo [64]. The few existing experiments involving 3D neuron–GB cocultures, including functional evaluation, were performed with rodent‐derived neurons [20, 23]. Here, we used Ca^2+^ imaging to show the functional activity of human‐derived neurons and GB cells in 3D. The Ca^2+^ signals detected from GB cells were comparable to those obtained from previous 2D cultures [8, 65]. Neuronal Ca^2+^ waves exhibited a typical pattern characterized by fast onset and slower decay [66] comparable to observations from previous in vitro studies [10, 31]. In comparison, the Ca^2+^ waves measured from GB cells were broader and slower, similar to the Ca^2+^ signal reportedly obtained from astrocytes [67]. These results indicate that the model environment supports the typical functionality of both cell types.

The presented Ca^2+^ analysis method is capable of diving deeper into cellular signaling, as it determines descriptive parameters from detected Ca^2+^ waves, opening the possibility of investigating potential functional changes and interactions that can result from GB cells integration into neuronal networks. Glioma cells can induce hyperexcitability in neurons, which can lead to brain tumor‐related seizures and epilepsy [44, 68], a phenomenon reported with Ca^2+^ imaging in vivo [64]. As translation from in vivo to in vitro, our results revealed different Ca^2+^ activity parameters for mono‐ and cocultured neurons, suggesting their adaptive behavior during interaction with GB cells. Interestingly, in a recent study, a hyperexcitatory response to kainic acid, a glutamate receptor antagonist, was associated with decreasing frequency and amplitude of Ca^2+^ oscillations in 3D human cortical neuronal networks [69]. Here, human cortical neurons depicted decreases in the same parameters in cocultures with GB, suggesting the formation of glutamatergic neuro‐GB synapses [7]. Conversely, neurons are known to promote Ca^2+^ activity in GB cells [23]. Here, the peak‐to‐peak distance and average amplitude of GB Ca^2+^ waves decreased in cocultures, indicative of neuron‐induced activation and the structural and electrical integration of GB cells into neuronal networks.

TMZ resistance in GB cells is affected by their culture environment, for example, 3D cultures promote drug resistance more than 2D cultures [70]. Similarly, cocultures with astrocytes, microglia, or macrophages have been shown to result in greater TMZ resistance than GB monocultures [13, 14, 71]. We observed a significant decrease in GB spheroid invasion rates with 400 μM TMZ, in line with the results of previous 3D models [13, 71]. Nevertheless, no significant difference in the TMZ response on spheroid growth in cocultures compared to GB controls was observed, indicating that human cortical neurons did not affect TMZ resistance in the utilized GB line. However, as there is a substantial patient‐specific heterogeneity between GBs [2], the effect of neurons on drug resistance needs to be evaluated in a personalized manner.

To our knowledge, this is the first study to assess the effects of TMZ on neuronal functionality in a 3D coculture model. While studying the GB drug response, it is crucial to simultaneously assess host tissue function because the brain is very sensitive to adverse seizurogenic effects. Previous studies have provided controversial results regarding the association of TMZ with neuronal hyperexcitability. While it has been shown to have inhibitory effects on glioma‐associated epilepsy [72], the FDA Adverse Event Reporting System includes adverse event reports of seizures due to TMZ treatment [73]. A recent study supported the use of 3D neuronal cultures in neuropharmacological safety screening based on functional Ca^2+^ oscillation responses to seizurogenic compounds with relatively high sensitivity [69]. Here, comparison of the Ca^2+^ activity of neurons in TMZ‐treated and untreated cocultures revealed no significant differences. TMZ at the concentration used did not cause substantial harm to neuronal networks or compromise cellular functionality, which was assumed as TMZ is a clinically used standard‐of‐care drug. Moreover, as recent research suggests the repurposing of antiseizure and antidepressant medicines for glioma treatment to disrupt neuron–glioma crosstalk [74], it will be important to test these medicines in models with cell types capable of responding to the therapy.

The presented model has its limitations. For example, the model contains only neural cells as a nonmalignant cell type, whereas the native glioma TME includes several cell types [16]. While examples of models containing even penta‐cultures have emerged [14, 36], increasing complexity challenges the current analysis methods. Although our approach provides advanced functional assessment, the segmentation method used to distinguish Ca^2+^ signals in cocultures might not result in 100% accuracy, as neurons can grow between GB cells. Nevertheless, our method demonstrated that the Ca^2+^ activity patterns of neurons changed in cocultures, indicative of cellular integration and interactions, while neurons did not respond to TMZ treatment. This study was performed with a single, well‐characterized GB cell line; therefore, it does not recapitulate the wide heterogeneity and behavior of GB tumors in general. The established method is applicable for introducing patient‐derived GB into the model in the future, enabling the evaluation of drug responses in personalized medicine approaches.

In conclusion, the developed 3D neuron–GB coculture model is viable, repeatable, and expresses several physiologically relevant features, providing a tool to explore integration and invasion of GB cells into their TME. It unravels neuron–GB interactions that affect cellular functionality and increases the credibility of drug response studies by also considering the safety aspect regarding nonmalignant brain cells.

## Author Contributions

Nanna Förster and Lotta Isosaari should be considered joint first authors. Nanna Förster, Lotta Isosaari, Oskari Kulta, Oona Junnila, Marjukka Pollari, Kirsi J. Rautajoki, and Susanna Narkilahti conceived and designed the research; Nanna Förster, Lotta Isosaari, Oskari Kulta, Oona Junnila, and Valtteri Vuolanto performed the research and acquired the data; Nanna Förster, Lotta Isosaari, Oskari Kulta, and Susanna Narkilahti analyzed and interpreted the data. Nanna Förster, Lotta Isosaari, and Oskari Kulta produced the figures. Nanna Förster, Lotta Isosaari, Oskari Kulta, and Susanna Narkilahti wrote the original draft of the manuscript. Kirsi J. Rautajoki and Susanna Narkilahti supervised the research. All authors were involved in revising the manuscript.

## Conflicts of Interest

The authors declare no conflicts of interest.

## Supporting information


**Video S1.** 3D visualization of neuron‐GB coculture model, related to Figure 1. A 3D visualization confirmed that cells were growing throughout the hydrogel and that GB cells did not only grow on top of the hydrogel but invaded into the structure. Blue = DAPI, Green = GFP, Red = S100β, Gray = MAP‐2 + βtub_III_. Scale bar is 150 μm.

**Video S2.** Example of spontaneous Ca^2+^ wave traveling in GB cells in 3D environment, related to Figure 4. Ca^2+^ oscillations in the cells were detected as intensity changes of a Ca^2+^ indicator Rhod‐4 (red). The video shows how a Ca^2+^ wave is propagating through the GB cell network. Scale bar is 50 μm.

**Video S3.** Example of spontaneous Ca^2+^ wave in a neuron in 3D environment, related to Figure 4. Ca^2+^ oscillations in the cells were detected as intensity changes of a Ca^2+^ indicator Rhod‐4 (red). Scale bar is 50 μm.

Text S1.

## Data Availability

The data that support the findings of this study are available in the Materials and Methods, Results, and/or Supporting Information S1 and Video S1–S3 of this article. Further information and requests for data, resources, and reagents should be directed to and will be fulfilled by the corresponding author.
