# Supplementary material for: Functional 3D Human Neuron–Glioblastoma Model Reveals Cellular Interactions Enabling Drug Safety Assessments
Source: FASEB J. 2025 Apr 25;39(8):e70567. doi: 10.1096/fj.202500291RR (PMC12023715; doi:10.1096/fj.202500291RR)
Supplement: Supplementary file 4 — Text S1. [file FSB2-39-e70567-s004.docx]

**This PDF file includes:**

Figures S1 to S6

Tables S1 to S3


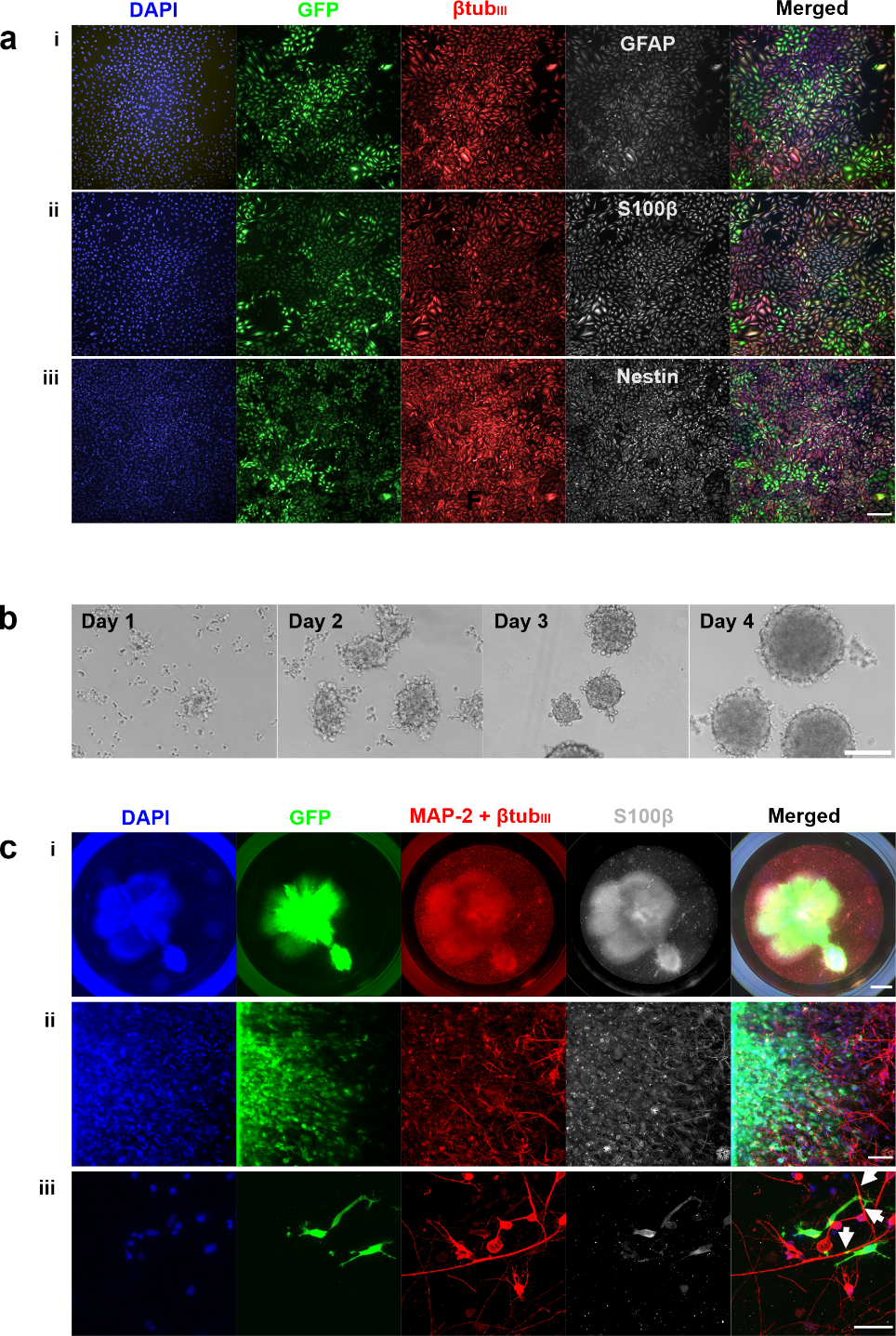


Figure S1. Characterization of LN229/GFP GB cells, related to Figure 1

a) In adherent conditions, GFP-labelled LN229/GFP cells showed characteristic morphology with mainly spindle-formed, triangular and some diamond shapes. LN229/GFP cells expressed all tested typical glial and neuronal markers: beta tubulin III (βtub_III_, neuronal marker, in red), glial fibrillary acidic protein (GFAP, glial marker, in gray in *i)*, S100 calcium-binding protein β (S100β, glial marker, in gray in *ii)*, and nestin (neural stem cell marker, in gray in *iii)*. Cell nuclei were stained with 4’,6-diamidino-2-phenylindole (DAPI). Scale bar is 200 µm. b) In non-adherent conditions, LN229/GFP cells formed different sized spheroids within 2-4 Days. Scale bar is 200 µm. c) Representative images of ICC staining of 3D neuron–GB cocultures show that cells stayed viable up to 4 weeks and hydrogels did not start degrading during that time. *i)* GB spheroids invaded most parts of the sample area which is not desired. Scale bar is 2000 µm. *ii)* Images from the neuron–GB border zone reveal that despite invading GB cells (GFP-labelled), neuronal networks stained with microtubule-associated protein 2 (MAP-2) and βtub_III_ remained intact. Scale bar is 100 µm. *iii)* Neurons and GB cells seemed to have similar contact points (shown by white arrows) as after 2 weeks of coculture, indicating that sufficient culture conditions can be maintained up to 4 weeks. Scale bar is 50 µm.


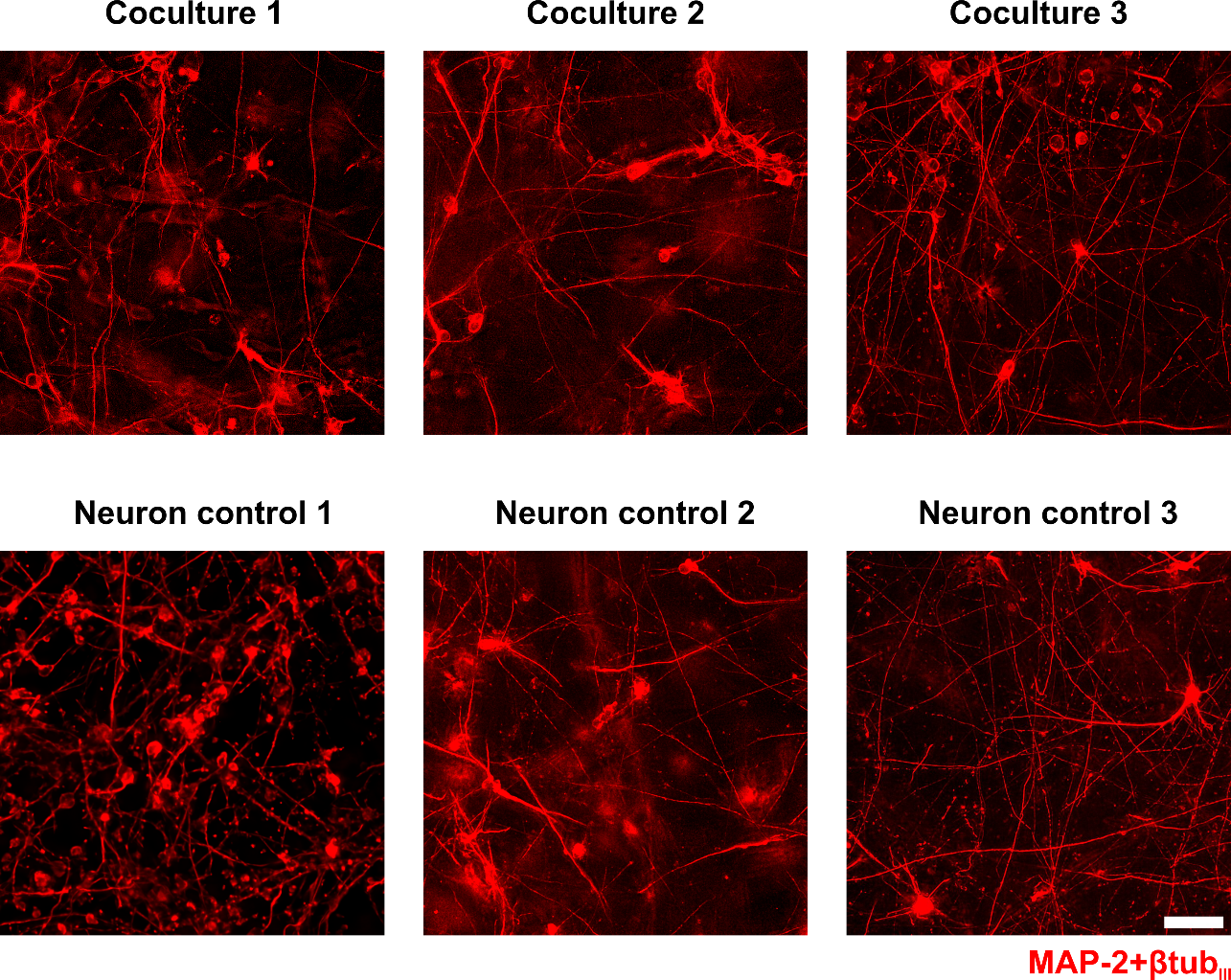


Figure S2. Neurons had similar morphology in cocultures and monocultures in 3D, related to Figure 2

ICC staining with MAP-2 + βtub_III_ revealed that all neurons 1, 2 and 3 expressed similar morphologies and formed similar networks in cocultures and neuron controls. Details of naming and numbering of the hiPSC lines underlying the neurons used in the experiments are listed in Table S1. Scale bar is 50 µm.


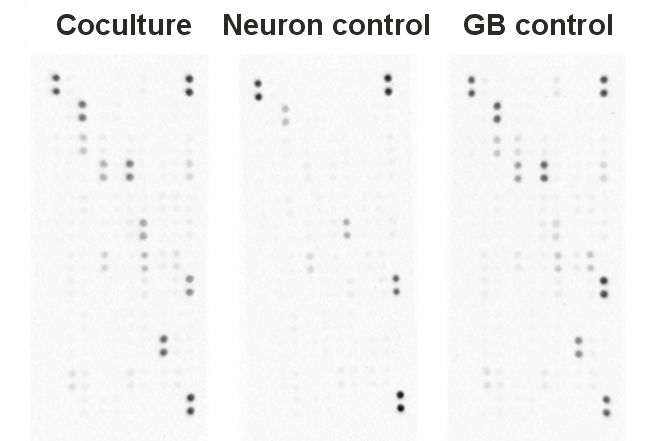


Figure S3. Proteome Profiler cytokine secretion data, related to Figure 3

Proteome profiler membranes from coculture, neuron and GB control used for the pixel density quantification. Medium samples were collected from experiment conducted with neurons derived from hiPSC line 1.


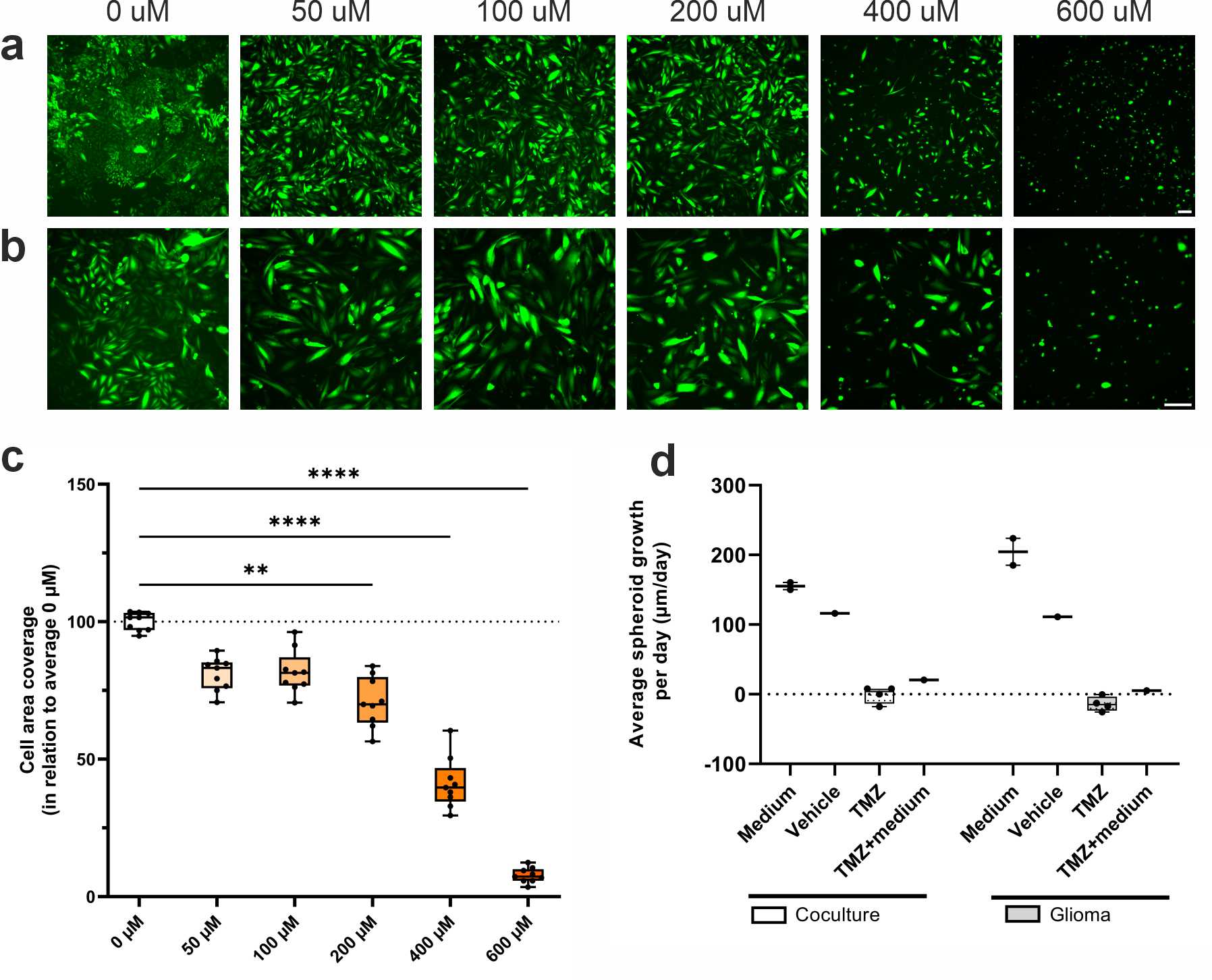


Figure S4. TMZ effect on LN229/GFP cells in 2D, and in 3D during 2. week after 400 µM TMZ treatment start, related to Figure 5

a) Different concentrations of TMZ were applied to 2D GB cell cultures for 7 Days. Already small concentrations of TMZ affected GB cell confluency. 50 µM, 100 µM and 200 µM TMZ conditions contain fewer cells compared to control condition (0 µM TMZ). A more dramatic difference was observed in samples treated with 400 µM or 600 µM TMZ where GB cells clearly started dying more. Scale bar is 200 µm. b) The larger the TMZ concentration was, the sparser the cells got. More round, most likely dead, cells were detected in high TMZ concentrations. Scale bar is 200 µm. c) Quantified cell area coverage percentages in relation to the 0 µM TMZ control. 200, 400 and 600 µM TMZ had significantly reduced cell area coverage compared to the control condition. Statistical significance was calculated with Kruskal–Wallis test with Dunn’s correction for multiple comparisons. d) Some samples were maintained after 7 Days of 400 µM TMZ treatment. Different conditions were tested for the following week: Medium = controls where both weeks in medium condition, Vehicle = controls where both weeks in 1 % DMSO medium condition, TMZ = both weeks in 400 µM TMZ condition, TMZ + medium = 7 Days of 400 µM TMZ treatment followed by 7 Days with only medium. Quantification of spheroid invasion rates (calculated from Day 21 to Day 28) revealed that two weeks of TMZ treatment stopped spheroid invasion completely. On the other hand, when culture conditions were after 7-Day TMZ treatment returned to medium condition, spheroid invasion seemed to start slowly again. For coculture conditions, $\boldsymbol{n}\left( \boldsymbol{Medium} \right)\boldsymbol{=2, n}\left( \boldsymbol{Vehicle} \right)\boldsymbol{=1, n}\left( \boldsymbol{TMZ} \right)\boldsymbol{=4, n}\left( \boldsymbol{TMZ+medium} \right)\boldsymbol{=1}$. For GB control conditions, $\boldsymbol{n}\left( \boldsymbol{Medium} \right)\boldsymbol{=2, n}\left( \boldsymbol{Vehicle} \right)\boldsymbol{=1, n}\left( \boldsymbol{TMZ} \right)\boldsymbol{=4, n}\left( \boldsymbol{TMZ+medium} \right)\boldsymbol{=1}$. The data is presented as a box plot, showing the mean and whiskers representing the minimum and maximum values.


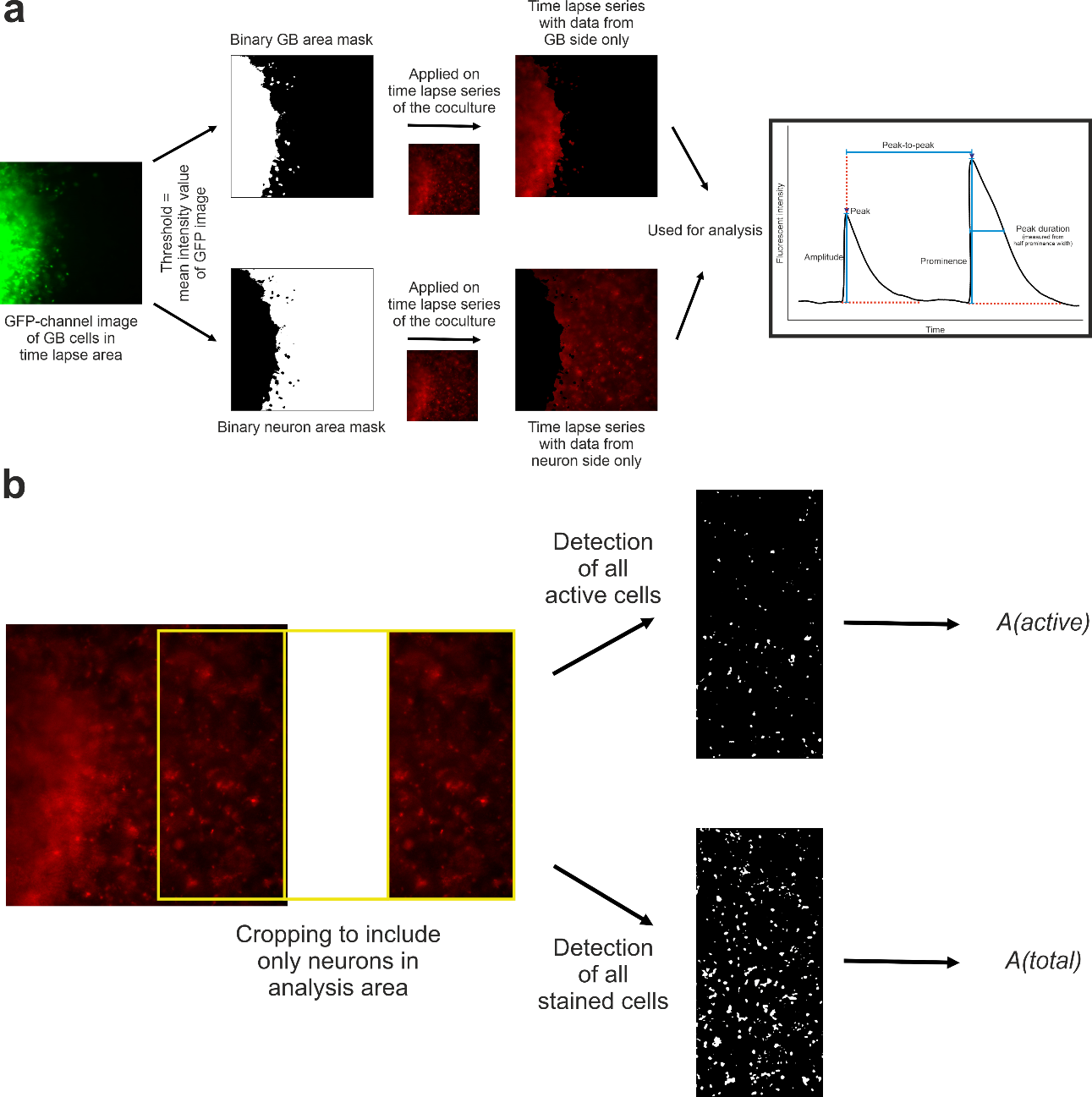


Figure S5. Workflow of calcium analysis approaches, related to Figure 6 and STAR Methods

a) Time lapse series of cocultures were segmented before analysis to differentiate between neurons and GB cells. Images of GFP-tagged (in green) GB cells were acquired in addition to time lapse series (in red). GFP-images were converted into binary images using their mean intensity as threshold value to get neuron and GB area masks. These masks were applied on coculture time lapse series to get GB sides and neuron sides separately for analysis. b) Percentage of active neuronal area was defined from cropped coculture time lapse series where areas with GB cells were excluded. From cropped regions, ImageJ was used to detect all active cell areas and all stained cell areas.


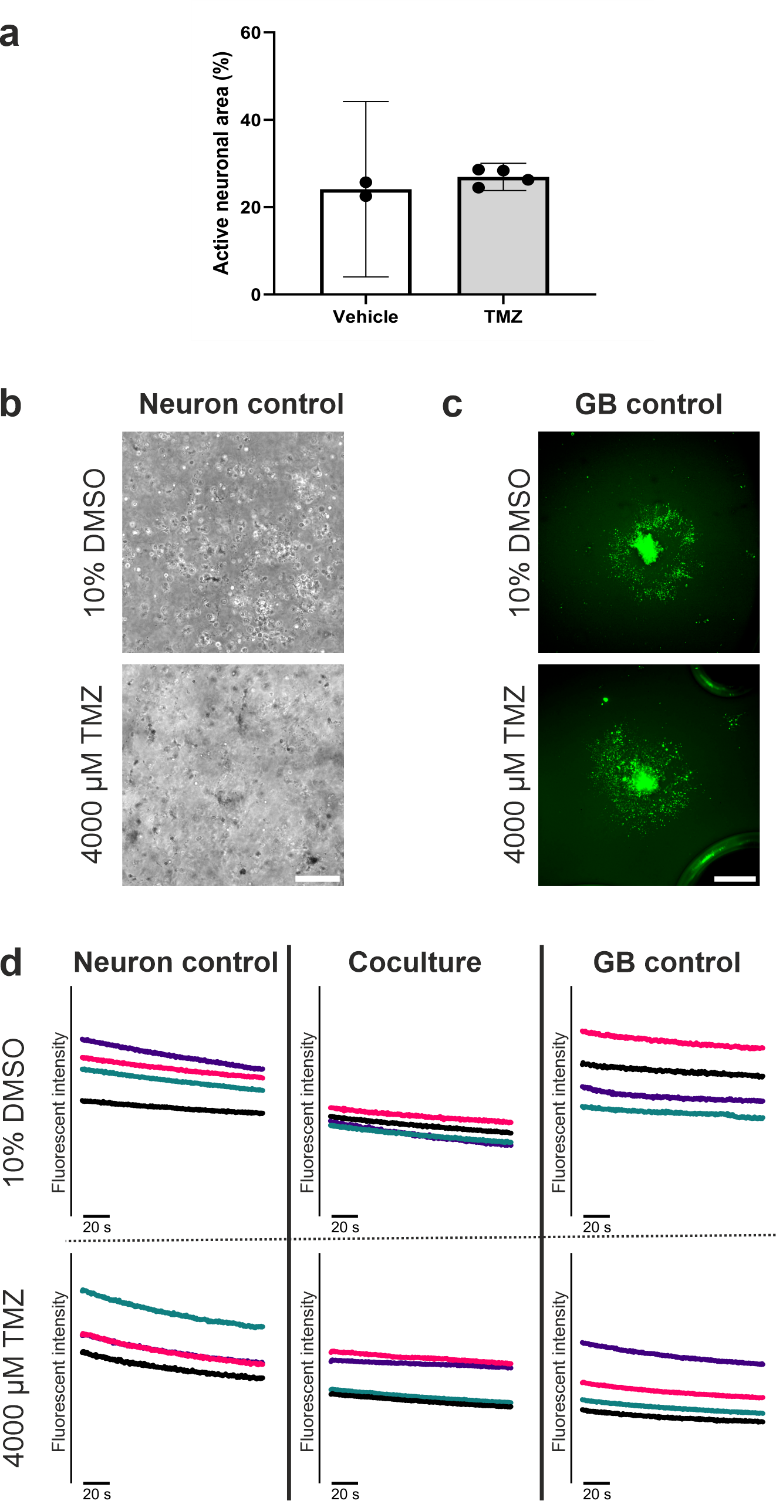


Figure S6. Effect of lower and higher TMZ concentration on neurons and GB cells in 3D, related to Figure 6

a) Active neuronal area percentage in cocultures did not alter under vehicle control (1 % DMSO) and 400 µM TMZ application. The data is presented as mean with 95% confidence interval. No significant differences were detected between the vehicle control ($\boldsymbol{n=3}$) and TMZ-treated ($\boldsymbol{n=4}$) groups. Neuron cell line 1 was used in the cocultures. b) Neurons did not look viable in 4000 µM TMZ or 10 % DMSO conditions. Neuronal morphology was changed: cell bodies turned into round shapes and neurite networks degraded and disappeared. Scale bar is 100 µm. c) GB cells did not look viable either in 4000 µM TMZ or 10 % DMSO conditions. GB spheroids broke down and cell morphology was mostly round. Scale bar is 1000 µm. d) No Ca^2+^ signals from 4000 µM TMZ treated samples nor from 10 % DMSO vehicle controls were detected, indicating that such high concentration is detrimental to cellular functionality.

**Table S1.** **All 105 proteins analyzed with the Proteome Profiler Human XL Cytokine Array Kit, related to Figure 3**

| Adiponectin/Acrp30 | IFN-gamma | CCL2/MCP-1 | GDF-15 |
| --- | --- | --- | --- |
| Angiogenin | IGFBP-2 | CCL7/MCP-3 | GM-CSF |
| Angiopoietin-1 | IGFBP-3 | M-CSF | CXCL1/GRO alpha |
| Angiopoietin-2 | IL-1 alpha/IL-1F1 | MIF | Growth Hormone (GH) |
| Apolipoprotein A1 | IL-1 beta/IL-1F2 | CXCL9/MIG | HGF |
| BAFF/BLyS/TNFSF13B | IL-1ra/IL-1F3 | CCL3/CCL4 MIP-1 alpha/beta | ICAM-1/CD54 |
| BDNF | IL-2 | CCL20/MIP-3 alpha | IL-27 |
| CD14 | IL-3 | CCL19/MIP-3 beta | IL-31 |
| CD30 | IL-4 | MMP-9 | IL-32 alpha/beta/gamma |
| CD31/PECAM-1 | IL-5 | Myeloperoxidase | IL-33 |
| CD40 Ligand/TNFSF5 | IL-6 | Osteopontin (OPN) | IL-34 |
| Chitinase 3-like | IL-8 | PDGF-AA | CXCL10/IP-10 |
| Complement Component C5/C5a | IL-10 | PDGF-AB/BB | CXCL11/I-TAC |
| Complement Factor D | IL-11 | Pentraxin 3/TSF-14 | Kallikrein 3/PSA |
| C-Reactive Protein/CRP | IL-12 p70 | CXCL4/PF4 | Leptin |
| Cripto-1 | IL-13 | RAGE | LIF |
| Cystatin C | IL-15 | CCL5/RANTES | Lipocalin-2/NGAL |
| Dkk-1 | IL-16 | RBP4 | Serpin E1/PAI-1 |
| DPPIV/CD26 | IL-17A | Relaxin-2 | SHBG |
| EGF | IL-18 BPa | TfR | ST2/IL1 R4 |
| CXCL5/ENA-78 | IL-19 | TGF-alpha | FGF basic |
| Endoglin/CD105 | IL-22 | Thrombospondin-1 | G-CSF |
| EMMPRIN | IL-23 | TIM-1 | FGF-19 |
| Fas Ligand | IL-24 | TNF-alpha | Vitamin D BP |
| uPAR | CCL17/TARC | Resistin | Flt-3 Ligand |
| VCAM-1 | TFF3 | CXCL12/SDF-1 alpha | KGF/FGF-7 |
| VEGF |  |  |  |

**Table S2.** **Sample size for each Ca^2+^ parameter and group related to Figure 4**

The presented *n* values stand for number of cells derived from specified number of individual recordings.

| **Ca^2+^ parameter** | **Neuron** | | | | **Coculture Neuron** | | | |
| --- | --- | --- | --- | --- | --- | --- | --- | --- |
|  | total *(from 8 record-ings)* | Neuron ID | | | total  *(from 13 record- ings)* | Neuron ID | | |
|  |  | 1 *(from 3 record-ings)* | 2 *(from 1 record-ing)* | 3 *(from 4 record-ings)* |  | 1  *(from 6 record-ings)* | 2  *(from 3 record-ings)* | 3  *(from 4 record-ings)* |
| **Average Amplitude** | 340 | 181 | 11 | 148 | 358 | 261 | 35 | 62 |
| **Average Prominence** | 341 | 182 | 11 | 148 | 359 | 261 | 35 | 62 |
| **Average Peak Width** | 341 | 182 | 11 | 148 | 359 | 261 | 34 | 63 |
| **Average Peak-to-Peak Distance** | 207 | 123 | 5 | 79 | 213 | 174 | 6 | 33 |
| **Slope Before Peaks** | 332 | 174 | 11 | 147 | 343 | 248 | 35 | 60 |
| **Slope After Peaks** | 334 | 177 | 11 | 146 | 347 | 250 | 35 | 62 |
| **Frequency** | 341 | | | | 341 | | | |

| **Ca^2+^ parameter** | **Coculture GB** | | | | **GB** |
| --- | --- | --- | --- | --- | --- |
|  | total  *(from 13 record-ings)* | Neuron ID | | | total  *(n cells from 7 recordings)* |
|  |  | 1  *(from 6 record-ings)* | 2  *(from 3 record-ings)* | 3  *(from 4 record-ings)* |  |
| **Average Amplitude** | 130 | 81 | 20 | 29 | 82 |
| **Average Prominence** | 131 | 81 | 21 | 28 | 82 |
| **Average Peak Width** | 131 | 81 | 20 | 29 | 16 |
| **Average Peak-to-Peak Distance** | 54 | 43 | 5 | 6 | 16 |
| **Slope Before Peaks** | 129 | 79 | 21 | 29 | 79 |
| **Slope After Peaks** | 126 | 77 | 20 | 29 | 78 |
| **Frequency** | 64 | | | | 82 |

**Table S3.** **Sample size for each Ca^2+^ parameter and group related to Figure 6**

The presented *n* values stand for number of cells derived from specified number of individual recordings.

| **Ca^2+^ Parameter** | **Coculture Neuron**  *(n cells from 2 recordings)* | **Coculture Neuron TMZ**  *(n cells from 6 recordings)* |
| --- | --- | --- |
| **Average Amplitude** | 33 | 119 |
| **Average Prominence** | 33 | 119 |
| **Average Peak Width** | 33 | 120 |
| **Average Peak-to-Peak Distance** | 15 | 46 |
| **Slope Before Peaks** | 33 | 120 |
| **Slope After Peaks** | 32 | 119 |
| **Frequency** | 54 | 211 |
